# Structural Mechanisms for VMAT2 inhibition by tetrabenazine

**DOI:** 10.1101/2023.09.05.556211

**Published:** 2023-09-05

**Authors:** Michael P. Dalton, Mary Hongying Cheng, Ivet Bahar, Jonathan A. Coleman

**Affiliations:** a. Department of Structural Biology, University of Pittsburgh, Pittsburgh, Pennsylvania 15213, USA.; b. Laufer Center for Physical and Quantitative Biology, and Department of Biochemistry and Cell Biology, School of Medicine, Stony Brook University, Stony Brook, NY 11794, USA

## Abstract

The vesicular monoamine transporter 2 (VMAT2) is a proton-dependent antiporter responsible for loading monoamine neurotransmitters into synaptic vesicles. Dysregulation of VMAT2 can lead to several neuropsychiatric disorders including Parkinson’s disease and schizophrenia. Furthermore, drugs such as amphetamine and MDMA are known to act on VMAT2, exemplifying its role in the mechanisms of actions for drugs of abuse. Despite VMAT2’s importance, there remains a critical lack of mechanistic understanding, largely driven by a lack of structural information. Here we report a 3.3 Å resolution cryo-EM structure of VMAT2 complexed with TBZ, a non-competitive inhibitor used in the treatment of Huntington’s chorea. We find TBZ interacts with residues in a central binding site, locking VMAT2 in an occluded conformation and providing a mechanistic basis for non-competitive inhibition. We further identify residues critical for intracellular and luminal gating, including a cluster of hydrophobic residues which are involved in a luminal gating strategy. Our structure also highlights three distinct polar networks that may determine VMAT2 conformational change and play a role in proton transduction. The structure elucidates mechanisms of VMAT2 inhibition and transport, providing insights into VMAT2 architecture, function, and the design of small-molecule therapeutics.

## Introduction

Neuronal signaling by monoaminergic neurotransmitters controls all aspects of human autonomic functions and behavior, and dysregulation of this leads to many neuropsychiatric diseases. Nearly 60 years ago^1,2^, secretory vesicles prepared from adrenal glands were shown to contain an activity that accumulated epinephrine, norepinephrine, and serotonin in an ATP-dependent manner^3^. Extensive characterization by many different laboratories of synaptic vesicles (SVs) in neurons showed that monoamine transport activity was also dependent on the proton gradient generated by the V-ATPase, exchanging two protons for one cationic monoamine^4–8^. Monoamine transport was shown to be inhibited by non-competitive inhibitors such as tetrabenazine (TBZ)^9^ and competitive inhibitors like reserpine which has been used to treat hypertension^3^. Amphetamines were shown to be monoamine transporter substrates, eventually leading to vesicle deacidification and dopamine release^10^. Cloning of the vesicular monoamine transporter (VMAT) in the 1990’s^11–13^ revealed two different genes, VMAT1 and VMAT2, that were expressed in the adrenal medulla and brain, respectively^12,14^. VMAT2 is expressed in all monoaminergic neurons in the brain, including those for serotonin, norepinephrine, dopamine, and histamine^3^, and is essential for loading these neurotransmitters into SVs. VMAT2 is fundamentally required for neurotransmitter recycling and release^15–17^ and changes in VMAT1 and VMAT2 activities either through small-molecule agents or mutations are thought to contribute to many human neuropsychiatric disorders including infantile-onset Parkinson’s, schizophrenia, alcoholism, autism, and bipolar depression^18–24^.

**Figure 1.**
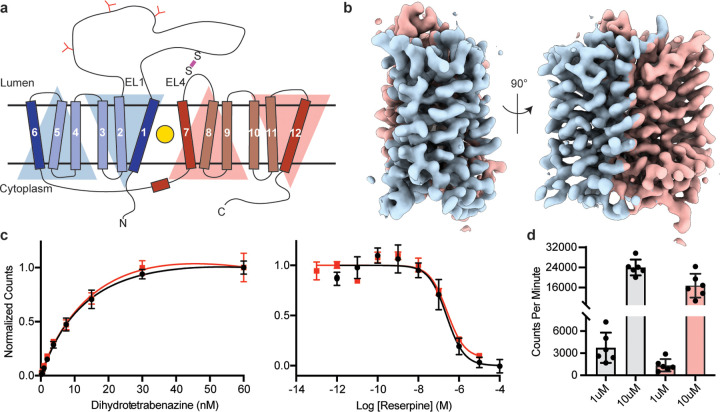
Cryo-EM reconstruction and functional characterization of VMAT2-tetrabenazine complex. **a,** Predicted structural elements of VMAT2. The neurotransmitter substrate is bound at the central site (yellow, triangle). The red and blue triangles depict the pseudo two-fold symmetric repeat comprised of TM1–6 and 7–12, respectively. A disulfide bond (purple line) is predicted between extracellular loop 1 (EL1) and EL4, N-linked glycosylation sites in EL1 are shown as red ‘Y’ shapes. **b,** Occluded map of VMAT2-tetrabenazine complex (3.3 Å resolution, contour level 0.076). The mVenus and GFP-Nb fiducial is not shown for clarity. **c,** Left panel, plots of [^3^H]dihydrotetrabenazine saturation binding to wild-type VMAT2 (black, circles) and chimera (red, squares). Symbols show the mean derived from n=3 technical replicates. Error bars show the s.e.m. Right panel, graphs of competition binding of ^3^H-dihydrotetrabenazine with unlabeled reserpine, error bars show the s.e.m. **d,** Plots of transport into vesicles using 1 and 10 µM ^3^H-serotonin for wild-type VMAT2 (grey bars) and chimera (red bars). The bars show the means and points show the value for each technical replicate. Error bars show the s.e.m.

VMAT1 and 2 are members of the solute carrier 18 (SLC18) family and are also known as SLC18A1 and SLC18A2. The SLC18 subfamily also includes the vesicular transporters for acetylcholine^25^ (VAChT, SLC18A3) and polyamines^26^ (VPAT, SLC18B1). Sequence alignments also show that SLC18 transporters belong to the major facilitator superfamily (MFS) of membrane transport proteins which use an alternating access mechanism^27,28^ to transport substrate across membranes. SLC18 members are predicted to be comprised of 12 transmembrane spanning helices (TMHs), which are arranged in two pseudosymmetric halves each with 6 TMHs containing a primary binding site for neurotransmitters, polyamines, and inhibitors located approximately halfway across the membrane^29^ (Fig. 1a). Conformational changes driven by the proton electrochemical gradient are thought to expose the binding site to both sides of the membrane allowing for transport of neurotransmitter from the cytoplasm to the lumen of SVs^30–32^.

VMAT2 and VMAT1 share 62% sequence identity but have distinct substrate specificity and pharmacological properties^33^. Small-molecule ligands such as TBZ and reserpine are high-affinity inhibitors of VMAT2, which prevent neurotransmitters from binding, arrest VMAT2 from cycling, and consequently reduce neuronal signaling. VMAT2 exhibits higher affinity for TBZ, as well as monoamines and amphetamines, whereas reserpine binds equally to both VMAT2 and VMAT1^9^. TBZ is the only drug which is approved for treatment of chorea associated with Huntington’s disease and has shown to be effective in various other hyperkinetic conditions such as tardive dyskinesia, dystonia, tics, and Tourette's syndrome^34^. A proposed mechanism for TBZ inhibition of VMAT2 involves two sequential steps, initial low-affinity binding of TBZ to the luminal-open state of VMAT2 which produces a conformational change, resulting in a high-affinity dead-end TBZ-bound occluded complex^30,35–37^.

Here we report a structure of VMAT2 bound to TBZ at 3.3 Å resolution in an occluded conformation using single-particle cryo-electron microscopy (cryo-EM), describing the architecture of VMAT2, identifying the high-affinity TBZ binding site, and revealing the mechanisms of drug and neurotransmitter binding, inhibition, and transport.

## Cryo-EM imaging of human VMAT2

Since VMAT2 is a small monomeric membrane protein of approximately 55 kDa, cryo-EM structure determination is challenging. To overcome this, we incorporated mVenus and the anti-GFP nanobody into the N- and C-terminus respectively of human VMAT2 to provide mass and molecular features to facilitate the cryo-EM reconstruction, this created a hook-like fiducial feature by reconstituting the interaction of these proteins on the cytosolic side of VMAT2^38^. Attachment of both proteins to the termini proved to be ineffective as the unstructured N- and C-terminus of VMAT2 are flexible; to combat this problem, we determined the minimal termini length that would reduce flexibility while maintaining VMAT2 folding. After successive optimizations, our final construct contained mVenus fused to the N-termini at position 18, and the anti-GFP nanobody at position 482 which we denote the VMAT2 chimera (ED Fig. 1a–d). We investigated the consequences of modification of VMAT2 in order to ensure the chimera maintained functional activity. First, we performed binding experiments with readily available ^3^H-labeled dihydrotetrabenazine (DTBZ) and found the chimera bound DTBZ with a similar affinity (K_d_ = 26 ± 9 nM) to the wild-type control (K_d_ = 18 ± 4 nM) (Fig. 1c). Competition binding of labeled DTBZ with unlabeled reserpine also produced a K_i_ 173 ± 1 nM which was similar to wild-type (161 ± 1 nM) (Fig. 1d). Next, we performed transport experiments using permeabilized cells, initial time course experiments using ^3^H-labeled serotonin showed clear accumulation (ED Fig. 1c), and steady-state experiments using 1 and 10 µM serotonin measured within the linear uptake range showed similar transport activity as wild-type VMAT2(Fig. 1e). Thus, the functional properties of the chimera are similar to the wild-type VMAT2.

To understand the architecture, locate the drug binding site, and how TBZ binding influences the conformation of the transporter, we studied the VMAT2 chimera using single-particle cryo-EM (ED Fig. 2). The resulting cryo-EM map was determined to a resolution of 3.3 Å, the TMH densities were well-defined, continuous, and exhibited density features for TBZ in the primary binding site and most of the side chains (ED Table 1, ED Fig. 3). This demonstrates the feasibility of our approach and enabled us to build a model of VMAT2.

## Architecture of VMAT2

The TBZ bound VMAT2 complex adopts an occluded conformation, with TBZ binding centrally in the transporter in a binding pocket located between the central transmembrane helices. The twelve TMHs of the transmembrane domain (TMD) of VMAT2 are arranged in a tight bundle with TMHs 1–6 and 7–12 each organized into a pseudosymmetrical half (Fig. 1b). The cytosolic facing side of VMAT2 is characterized primarily by the unstructured N- and C-termini along with a 20-residue loop that connects the two halves, extending from TM6 to TM7 before terminating in a short alpha helix that runs parallel to the bilayer and connects to TM7 with a short linker. TM5 and 11 both contain proline residues near the luminal face, which break the helical structure and facilitate connections to TM6 and 12 respectively. TM9 and 12 exhibit significant heterogeneity in our cryo-EM reconstructions, we speculate that this is likely due to a dynamic nature intrinsic to the TMs, an aspect that may offer a glimpse into VMAT2 dynamics.

VMAT1 and 2 encode a large extracellular loop (EL) 1 which contains several N-linked glycosylation sites^39^ and a disulfide bridge between EL1 and EL4^40^. EL1 and 4 also contain intriguing elements of structure, EL1 extends into the luminal space in an unstructured loop which is mostly not resolved in our structure, before terminating in short helix which interacts with the luminal face of the transporter near TM7, 11 and 12. EL4 extends outward from TM7 into the lumen before connecting back to TM8. A striking feature of EL4 is the location of W318 which positions its indole side chain directly into a luminal cavity near the TBZ site, acting as a plug to completely occlude the luminal side of the transporter. Together, these loops cinch the luminal side of the transporter closed, locking VMAT2 in an occluded conformation and preventing ligand egress. The conserved nature of EL4 and W318 suggests this motif is necessary for transport function and is a key player in the transporter mechanism (ED Fig. 1d). The conformation of EL1 and 4 is likely also aided by a disulfide bond between cysteines 126 and 333, which is known to be necessary for transporter function^40^. While our structure was not able to unequivocally place this bond due to the lack of density for residues 48–118 of EL1, the disulfide likely restricts the movement of EL4 which orders this loop in a more rigid state.

Comparison of the transmembrane domain with more distantly related MFS transporters in other conformational states such as the outward-open VGLUT2^41^ (PDB code 8SBE) and inward-open GLUT4^42^ (7WSM) models (1.2 Å root-mean-square-deviation, RMSD) show that conformational changes involving TMs 1, 7, 8 and 11 are likely involved in mediating the transport cycle and alternating access (ED Fig. 6). The conformation of the TMD of VMAT2 largely resembles that predicted by Alphafold^43^ with a 1.1 Å RMSD overall difference in the transmembrane domain. However, Alphafold lacks several key features such as in the position of the ELs and is unable to predict key details that are critical for ligand binding. Hence, computational docking could not capture a stable TBZ bound state using Alphafold, alluding to the critical importance of our experimental structure in understanding of VMAT2 molecular mechanisms.

Mutation of P316A, P237H, and P387L in VMAT2 abolishes monoamine transport, causing an infantile-onset form of parkinsonism^44–46^, we find that these residues are located in EL4 and in the luminal ends of TM5 and 9 respectively (ED Fig. 5d). EL4 is involved in luminal gating and the P316A variant would likely disrupt the conformation of the loop. In the case of proline 237, a histidine would result in not only an insertion of a positively charged residue into the luminal membrane interface but would also reduce the helical bend and distort the connection of TM5 with TM6. The P387L variant would also disrupt helical connections and the overall architecture of the helix by insertion of a bulky residue into a small hydrophobic cavity. Therefore, these SNPs likely alter the ability of the transporter to sample multiple conformations by reducing transporter dynamics and also perturb VMAT2 folding. Recently, many additional disease variants have been discovered^47^ many of which are also found in the luminal or cytoplasmic ends of TMHs, EL1, and the N- and C-termini.

## Intracellular and luminal gates

**Figure 2.**
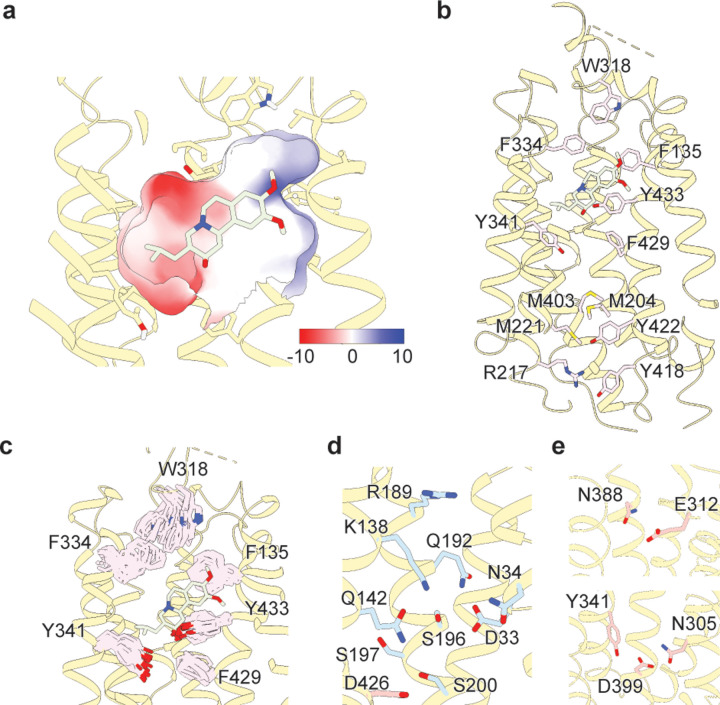
VMAT2 conformation and residues involved in gating. **a,** ‘slice view” through an electrostatic surface representation of the VMAT2-tetrabenazine (TBZ) complex. TBZ is shown in light green sticks. **b,** Cartoon representation showing the extracellular gating residues and the intracellular gating residues in pink sticks. **c,** Variations of gating residue poses captured in molecular dynamics simulations. **d,** Cartoon representation of polar network ‘one’. Blue and red sticks denote residues in the N- and C-terminal half respectively. **e**, Polar network ‘two’ (top) and ‘three’ (bottom), residues colored in red.

The structure of the VMAT2-TBZ complex reveals that both the cytosolic and luminal gates are closed which precludes solvent and ligand access from either the cytosolic or luminal compartments (Fig. 2a,b). Previous studies have suggested residues R217, M221, Y418 and Y422 make up the cytosolic gate^32^, we find R217 and Y418 form the outer cytosolic gate with the guanidino group of R217 involved in a cation-pi interaction with the aromatic benzyl group of Y418 which seals off intracellular access to the binding site. M221 and Y422 form a second set of intracellular gating residues ‘above’ the outer cytoplasmic gate through a stable methionine-aromatic interaction which acts to fully seal the cytoplasmic gate. It is likely that M204 and M403 also contribute to cytosolic gating in this region as their side chains also act to fill this space. On the luminal side, F135, F334 and W318, form the luminal gate where they interact with one another to block access to the binding site. W318 acts as ‘cap’ with the indole side chain facing into a tightly packed hydrophobic pocket consisting of residues I44, V131, L134, I315, I317, and I381 which completely prevent access on the luminal side. W318 is highly conserved in the SLC18 family, suggesting that SLC18 transporters share a common mechanism of luminal-gate closure (ED Fig 5c). E127 of EL1 may play a role in stabilizing the tryptophan in this conformation, with the carboxyl group of the side chain orienting itself near the indole nitrogen potentially forming a hydrogen bond pair. The inner gate is located ‘below’ the TBZ ligand and involves residues Y341, F429, and Y433^32^. The large aromatic side chains of these residues may compartmentalize the transporter, ensuring directional transport of substrate. MD simulations revealed little variation in the pose of the aromatic gating residues comprising the inner gates (Fig. 2c). However, the luminal gates showed more movement, likely owing to its residue composition and lack of strong interactions. Despite this observation, we would assert that the tight hydrophobic environment likely prevents exchange from the luminal space. These mechanisms of gating are atypical of MFS transporters which more commonly use salt bridges to gate access to the substrate binding site^48^.

## Polar networks

Upon careful inspection of the model, we were able to identify distinct polar networks that we believe may play a role in proton coordination and subsequent transporter conformational change. The first and largest of these networks lies between TMs 1, 4 and 11, and consists of residues D33, N34, K138, Q142, R189, Q192, S196, S197, S200 and D426 (Fig. 2d)^31^. At the center of this network lies D33^32^, which makes critical contacts with the side chains of N34, K138, S196, and Q192. Together, the residues comprise a complex hydrogen bond network linking TM 1, 4 and 11. D426^49^ lies further toward the cytosol with the side chain carboxyl group facing the bulk of the network, likely forming a hydrogen bond with the hydroxyl group of S200. In the other TMD half there are two distinct groups of interacting polar residues, which bridge between TM 7, 8 and 10 (Fig 2e). The second group is a pair of residues found on the luminal side, between residues E312 and N388 with the amide group of the N388 side chain pointed towards the carboxyl group of E312, which could act to stabilize TBZ in the binding site. The third group is located toward the cytosolic side and consists of N305, Y341, and D399^49^, the latter two of which have previously been speculated to form a hydrogen bond pair^31^. The side chains of these residues are positioned toward one another, with the carboxyl group of D399 forming a hydrogen bond with N305 and likely Y314. Taken together, we believe these networks play a critical role in mediating conformation change in the transporter. We hypothesize that protonation of D33, E312, and D399 would greatly perturb these interactions by breaking crucial hydrogen bond pairs, leading to opening of the cytosolic gate. The asymmetry between these two networks is also striking, with the first network consisting of TMs 1, 4 and 11 being substantially larger. This may allow for larger conformational changes on this side and an overall asymmetry in the cytosolic-open state of the transporter. To our knowledge this is an atypical feature in MFS proteins^48^ and would represent an interesting adaptation upon the rocker-switch mechanism.

## Tetrabenazine binding site

**Figure 3.**
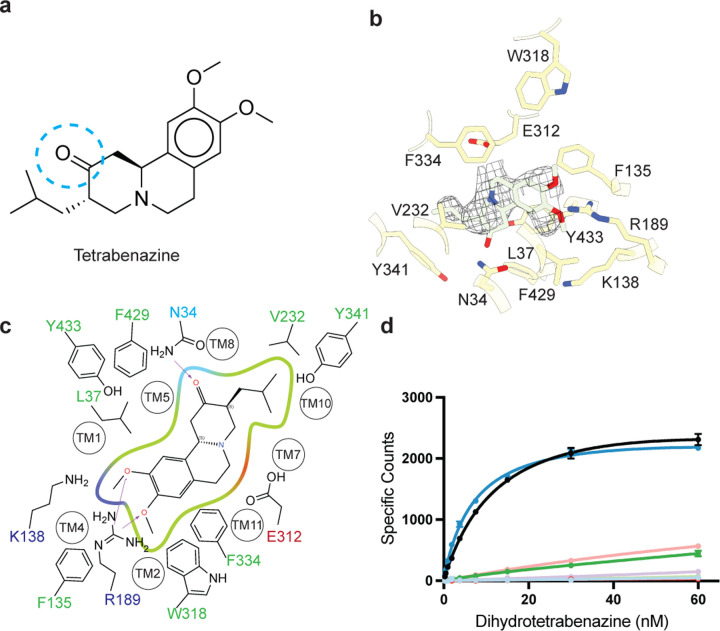
Tetrabenazine recognition and binding. **a,** Chemical structure of tetrabenazine (TBZ). The blue dotted circle indicates the position of the hydroxyl group in dihydrotetrabenazine. **b,** Binding site of TBZ, residues which are involved in binding are shown in tan sticks. TBZ is shown in light green sticks and the associated density in dark grey mesh. c, 2D cartoon of the TBZ binding site showing only highlighted residues. Green, red, and blue indicate hydrophobic, negative, or positively charged properties of the side chains. **d,** Plots of ^3^H-dihydrotetrabenazine saturation binding to wild-type (black line) and mutant VMAT2. F135A (light blue), R189A (red), V232L (dark green), E312Q (salmon), W318A (light purple), F429A (dark blue), and Y433A (light green). Symbols show the mean derived from n=3 technical replicates. Error bars show the s.e.m.

TBZ (Fig. 3a) adopts a pose which is predominantly perpendicular to the direction of the TMHs in the luminal half of VMAT2 just ‘below’ the location of the luminal gating residues. The TBZ binding site exhibits an amphipathic environment, comprised of both polar and non-polar residues (Fig. 2a, Fig. 3c). The tertiary amine of TBZ orients itself towards the negatively charged surface of the binding site near TMs 7 and 11, and toward E312 (Fig. 3b). E312 plays an analogous role to the highly conserved aspartate residue present in neurotransmitter sodium symporters^50^ which also is involved in directly binding to amine groups (ED Fig. 7). Because E312 was previously shown to be necessary for substrate transport and inhibitor binding, we first selected this residue for mutagenesis to probe its importance in TBZ binding^31,51^. We performed radiolabeled binding experiments to assess the effect of mutating residues in the TBZ binding site by measuring binding of ^3^H-labeled DTBZ (Fig. 3d). Mutation of E312Q does not fully abolish DTBZ binding, suggesting that it may not be essential for TBZ binding (ED Fig. 5b), however the E312Q mutant greatly reduced DTBZ affinity, demonstrating that, while not completely essential, it still remains important in inhibitor binding and likely substrate transport by interacting with the amine of the neurotransmitter (Fig. 3b). Next, we observed that R189 orients the guanidino group towards the methoxy groups of TBZ likely forming hydrogen-bonding interactions and we found that replacement of R189 with an alanine nearly completely abolished DBTZ binding at all concentrations tested (Fig. 3d, ED Fig. 5b). We speculate that R189 may also be involved substrate transport, by forming interactions with the hydroxyl groups of dopamine or serotonin (ED Fig. 7). Lysine 138, has been previously shown to play an important role in both TBZ binding and serotonin transport, and positions the primary amine side chain toward the methoxy groups of TBZ^49^ (Fig. 3b). K138 is positioned between two aspartate residues (D426 and D33) and is part of a hydrogen bond network that has been previously hypothesized^49^. Previous experiments found that mutating K138 to alanine resulted in an approximate 4-fold reduction in TBZ binding affinity^31^. While significant, this did not abolish TBZ binding and K138 may play a more significant role in inducing conformational changes during proton transport rather than TBZ binding. Asparagine 34 is of particular interest since the amide group of the sidechain of N34 appears to form a hydrogen bond with the carbonyl oxygen of TBZ (Fig 3b). DTBZ is a metabolite of TBZ^52^ which differs by only a hydroxyl group *vs.* a double bonded oxygen and binds to VMAT2 with a modestly higher affinity^35,53^ (Fig. 3a). Our structure suggests that the amide of N34 acts as a hydrogen bond donor for TBZ, and in the case of DTBZ the hydroxyl of the ligand may act as a hydrogen bond donor for the carbonyl oxygen of N34. We hypothesize that this interaction is more favorable for DTBZ, leading to a higher binding affinity. Valbenazine, is a TBZ analogue with a valine attached to the oxygen of the hydroxyl group of DTBZ and binds VMAT2 with an affinity of 150 nM^54^. We hypothesize that N34 does not form a favorable hydrogen bond with the oxygen of valbenazine and that addition of this larger moiety causes steric clashes in the binding site.

Large hydrophobic residues make a significant number of contacts with TBZ in the binding site and act as both space-filling residues and form critical aromatic interactions with the ligand. F135, Y433, and W318 all reduce DTBZ binding when mutated to alanine (Fig. 3b, 3c). Extensive contacts of TBZ with F135 may function to keep the transporter closed on the luminal side, which would trap VMAT2 in the occluded conformation. F135 and Y433 form pi-stacking interactions with TBZ which coordinate the benzene ring of TBZ. F429A did not reduce DTBZ affinity (K_d_ = 7.7 ± 0.6 nM) compared to the wild-type control (K_d_ = 15 ± 2 nM), revealing that although mutation of this residue would compromise the inner cytosolic gate, it is not directly involved in binding TBZ. Conversely, while W318 in EL4 also does not interact directly with TBZ, W318 is required for stabilizing the occluded conformation, and replacement with alanine prevents TBZ from being trapped inside the transporter by preventing closure of the luminal gate. Mutation of the disulfide bond between EL1 and 4 plays a critical role in transport^40^, and the disulfide may function to restrict the dynamics of this region to allow W318 to occlude the neurotransmitter binding site during transport.

Our model of the VMAT2-TBZ complex allowed us to pinpoint two residues which contribute to the specificity of TBZ to VMAT2 over VMAT1. Previous studies have highlighted V232^51^, which is a leucine in VMAT1, as being putatively involved in conferring differences in affinity, and our model shows that V232 is positioned closely to the isobutyl of TBZ which is wedged into a small hydrophobic pocket (ED Fig. 5a). The addition of an extra carbon of the leucine sidechain would produce a steric clash and limit the ability of TBZ to bind (Fig. 2b). The V232L mutant in VMAT2 reduces the affinity of DTBZ to VMAT2, confirming its importance in specificity, but the V232L mutant did not show a complete loss in binding (Fig. 3d, ED Fig. 5b). Therefore, we carefully inspected the binding site of VMAT2 and compared it to the predicted structure of VMAT1 to find additional differences in the binding site, we found that L37 in VMAT2 is a phenylalanine in VMAT1. Given its proximity to TBZ, this substitution would produce a steric clash with the benzene ring (ED Fig. 5a). Although the L37F mutant was expressed poorly and we could not evaluate the entire DTBZ concentration range, binding at 60 nM DTBZ was reduced to nearly zero (ED Fig. 5b). Thus, we believe the combination of these two substitutions constitute the differences in TBZ affinity of VMAT2 *vs.* VMAT1.

Molecular dynamics simulations suggest that there are two distinct binding poses of TBZ (ED Fig. 4). The predominant pose in our simulations is identical to the pose resolved in our cryo-EM structure (0.4 Å RMSD). In the second pose (3 Å RMSD), the methoxy groups of TBZ orient themselves toward cysteine 430, a residue previously identified to play an important role in binding TBZ^55^. We believe this pose provides insight into the mechanism by which TBZ enters and eventually positions itself into the pose resolved in our cryo-EM map. TBZ is thought to enter from the luminal side of VMAT2 by binding to the luminal-open conformation^37^, TBZ may interact first with C430 and the other residues of this initial pose before R189 moves between the two methoxy groups and allows TBZ to settle into the resolved orientation (ED Fig. 4e, ED Fig. 4f). This result highlights the stepwise process inhibitors like TBZ undergo to bind their targets.

## Mechanism of TBZ inhibition and neurotransmitter transport

**Figure 4.**
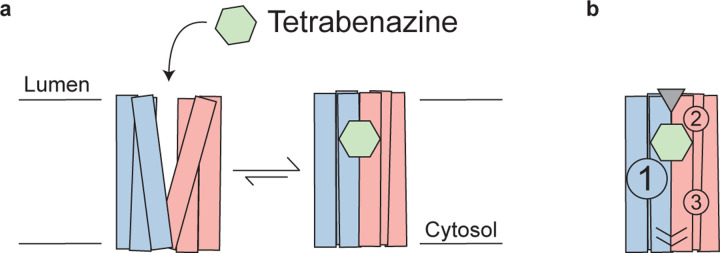
Mechanism of tetrabenazine inhibition, gating mechanisms, and polar networks. **a,** Cartoon depicting tetrabenazine binding to VMAT2. Tetrabenazine (green hexagon) binds to the luminal-facing state and induces conformational change to a high-affinity occluded conformation which is the resolved cryo-EM structure reported in this work. **b,** Cartoon model of the VMAT2 – tetrabenazine complex highlighting significant features including both cytosolic (slashes) and luminal gates (triangle), the three polar networks (numbered circles) and relative location of the tetrabenazine binding site (green hexagon).

The VMAT2 – TBZ complex captures the transporter in a fully occluded state with centrally located ligand binding site. VMAT2 functions by alternating access which involves exposure of the primary binding site to both sides of the membrane and isomerization between a cytosolic-open and luminal-open state in a mechanism known as the rocker-switch (Fig 4a)^3,29,48^. Since TBZ is a non-competitive inhibitor of neurotransmitter transporter, it enters VMAT2 from the luminal side, binding to a luminal-open conformation (Fig 4a)^35^. TBZ makes extensive contacts with residues in the primary site, likely in a lower affinity state as the transporter subsequently closes to form the high-affinity occluded state (Fig. 4b). The luminal gates lock the transporter into an occluded state, preventing displacement by other ligands and producing a so-called dead-end complex^32,34,35,52^. Our structure also provides important clues for understanding the chemical specificity and selectivity of TBZ binding, suggesting that the enhanced affinity of DTBZ is due to preferential interaction with N34. Comparison of the residues involved in TBZ binding in VMAT2 *vs.* VMAT1 also provides insight into the selectivity of TBZ by demonstrating that key differences in the ligand binding site are likely responsible for the reduction in TBZ binding affinity observed in VMAT1^9^. We also highlight three key polar networks which may be involved in conformational changes induced by proton binding during the transport cycle and are likely also involved in mediating proton transduction. Thus, our work provides a framework for understanding the structural underpinnings of neurotransmitter transport and inhibition in VMAT2 and other related transport proteins.

## METHODS

### Data reporting

No statistical methods were used to predetermine sample size. The experiments were not randomized and the investigators were not blinded to allocation during experiments and outcome assessment.

### VMAT2 construct design and cloning

Wild-type VMAT2 was expressed as a C-terminal eGFP fusion protein containing an 8x His-tag. The VMAT2 chimera consisted of mVenus^56,57^ fused to the N-terminus of VMAT2 at amino acid position 18, and the anti-GFP nanobody^38,57^ containing both a 10x His-tag and a TwinStrep tag fused to the C-terminus at position 482 by Infusion cloning. Single point mutants were made using PCR starting from wild-type VMAT2 C-terminally tagged eGFP construct, and constructs were initially evaluated using FSEC^58^.

### Expression and purification

VMAT2 was expressed in HEK293S GnTI^−^ cells^59^ using baculovirus mediated transduction^60^. Enriched membranes were first isolated by sonication followed by an initial spin at 10,000g followed by a 100,000g spin and subsequent homogenization. Membranes resuspended in 25 mM Tris pH 8.0 and 150 mM NaCl and frozen at −80°C until use. Membranes were thawed and solubilized in 20 mM n-Dodecyl-β-D-maltoside (DDM) and 2.5 mM cholesteryl hemisuccinate (CHS) with 1 mM DTT and 10 µM TBZ for 1 hr before centrifugation at 100,000g. VMAT2 was purified into buffer containing 1 mM DDM, 0.125 mM CHS, 25 mM Tris, 150 mM NaCl, 1 mM DTT, and 1 µM TBZ pH 8.0 using either a NiNTA column which was eluted in the same buffer containing 250 mM imidazole (for the wild-type VMAT2 C-terminally tagged eGFP protein) or a StrepTactin column eluted with 5 mM desthiobiotin. Purified VMAT2 was pooled and concentrated using a 100 kDa concentrator (Amicon) before separating by size-exclusion chromatography on a Superose 6 Increase column in 1 mM DDM, 0.125 mM CHS, 25 mM Tris pH 8.0, 150 mM NaCl, 1 mM DTT, and 1 µM TBZ. Peak fractions were pooled, concentrated to 6 mg/ml with a 100 kDa concentrator before addition of 100 µM TBZ, and ultracentrifuged at 60,000g prior to cryo-EM grid preparation.

### Cryo-EM sample preparation and data acquisition

The VMAT2 chimera (1.5 µl) at a concentration of 6 mg/ml was applied to glow discharged Quantifoil holey carbon grids (1.2/1.3 or 2/1 200 mesh gold or copper). Grids were blotted for 4 seconds at 100% humidity, 4°C, with a blot force of 4 using a Vitrobot Mk IV (ThermoFisher) before flash freezing into a 50/50 mixture of liquid propane/ethane cooled to ~-170°C with liquid nitrogen. Movies containing 40 frames were recorded on a FEI Titan Krios operating at 300 kV equipped with a Gatan K3 direct electron detector and a Bioquantum energy filter set to a slit width of 20 eV. Images were collected in super-resolution counting mode at a pixel size of 0.647 Å/pixel with defocus ranges from −1 to −2.5 um with a total dose of 60 e/Å^2^. Images were recorded using SerialEM^61^.

### Image processing

Micrographs were corrected using Patch Motion Correction and contrast transfer function estimated using Patch CTF in CryoSPARC v4.2^62^. A total of 24,875 micrographs were collected between two datasets recorded on the same microscope. Particles were initially classified by 2D classification in CryoSPARC to generate an ab-initio model for template picking which resulted in ~10 million picks which were extracted at a box size of 384 binned to 128 and classified multiple times using 2D classification and heterorefinement using a newly generated ab-initio model, an empty detergent ‘decoy’ class, and a junk class containing random density. The resulting approximately 500,000 particles from each dataset were refined using non-uniform refinement^63^, and combined before being subjected to further classification and refinement. Particles were refined locally using a mask including the TMD and the fiducial, followed by local refinement with a mask only including the TMD. Particles were subjected to local CTF refinement using the TMD mask before successive rounds of classification. 216,224 particles were then refined and subjected to Bayesian polishing in RELION 3.1^64^. The resulting 3.4 Å map still exhibited significant anisotropy, and was subjected to further rounds of classification and refinement with a TMD mask to improve features of the peripheral TMs. The resulting 3.3 A map was sharpened in DeepEMhancer^65^ using the highRes model and noise statistics settings features.

### Model building

The resulting EM map was sufficient for modeling most VMAT2 sidechains in the TMD. The Alphafold2^43^ model of VMAT2 was used for initial fitting and was further refined using RosettaCM^66^. After successive rounds of RosettaCM, the model was locally fit using Coot 0.98^67^ and Isolde^68^ with the majority of the manual rebuilding being done in Isolde. The model was refined in real space using Phenix 1.2^69^ and validated by comparing the FSC between the half maps and the refined model.

### Radioligand binding assays

Purified VMAT2 (wild-type eGFP tagged and VMAT2 chimera) were diluted to 5 nM in 1 mM DDM, 0.125 mM CHS in 20 mM Tris pH 8.0, 150 mM NaCl with 1 mg/ml CuYSi beads (Perkin Elmer). Protein was then mixed 1:1 to a final protein concentration of 2.5 nM in detergent buffer containing serially diluted ^3^H-labeled DTBZ (American Radiolabeled Chemicals) starting at 60 nM final concentration. Counts were then measured using a Microbeta2 scintillation counter in 96 well plates with triplicate measurements^70^. Specific counts were obtained by subtracting values obtained by the addition of 100 µM reserpine. Mutants were evaluated similarly from cell lysates of transfected cells. Data were fit to a single-site binding equation using Graphpad Prism.

Competition binding experiments were performed at the same protein concentration in the same detergent buffer. 10 nM ^3^H-labeled DTBZ was added to buffer and used for nine 1:1 serial dilutions with detergent buffer which initially contained 100 µM reserpine (10 µM for chimera). Measurements were done in triplicates and fit with a one-site competitive binding equation in Graphpad Prism.

### Serotonin transport

Cells transduced overnight were spun down and resuspended in 140 mM KCl, 5 mM MgCl_2_, 50 mM HEPES-KOH pH 7.4 and 0.3 M sucrose. Cells were permeabilized at 30°C for 10 min in the presence of 5 mM MgATP and 0.01% digitonin^32^. Controls additionally included 100 µM reserpine. After 10 min, cells were spun down and resuspended in the same buffer containing 2.5 mM MgATP and incubated at 30°C for 10 min. Cells were then mixed 1:1 with buffer containing ^3^H-labeled serotonin at a final concentration of 1 or 10 µM and incubated at room temperature for 6 min. Transport was stopped by the addition of ice-cold buffer, and the cells were collected on Glass Fiber C filters. The filters were then counted in scintillation fluid. Time course experiments were performed in the same way using 1 µM serotonin.

### Molecular dynamics simulations

The initial MD simulation system was prepared using CHARMM-GUI Membrane Builder module^71^. The structure of VMAT2 bound to TBZ was aligned using PPM2.0 webserver^72^ and embedded into 1-palmitoyl-2-oleoyl-sn-glycero-3-phosphocholine (POPC) membrane lipids. The protonation states of titratable residues of VMAT2 were assigned based on pKa calculation using PROPKA 3.0^73^; in particular E312 and D399 were protonated. TIP3P waters and K^+^ and Cl^−^ ions corresponding to 0.1 M KCl solution were added to build a simulation box of 92 ×92 ×108 Å^3^. The simulated system contained approximately 86,000 atoms, including VMAT2 and TBZ, 203 POPC molecules, 30 potassium (K^+^) and 36 chloride (Cl^−^) ions and 17,400 water molecules.

All MD simulations were performed using NAMD^74^ (version NAMD_2.13), following default protocol and parameters implemented in CHARMM-GUI^75^. Briefly, CHARMM36 force fields were adopted for VMAT2, lipids, and water molecules^76,77^. Force field parameters for TBZ were obtained from the CHARMM General Force Field for drug-like molecules^78^. Prior to productive runs, the simulation system was energy-minimized for 10,000 steps, followed by 2 ns Nosé–Hoover^79,80^ constant pressure (1 bar, 310 K; NPT) simulation during which the constraints on the protein backbone were reduced from 10 to 0 kcal/mol. Finally, the unconstrained protein was subjected to 100 ns NPT simulations. Periodic boundary conditions were employed for all simulations, and the particle mesh Ewald (PME) method^81^ was used for long-range electrostatic interactions with the pair list distance of 16.0 Å. The simulation time step was set to 2 fs with the covalent hydrogen bonds constrained with the SHAKE algorithm^82^. A force-based switching function was used for Lennard-Jones interactions with switching distance set to 10 Å. Langevin dynamics was applied with a piston period of 50 fs and a piston decay of 25 fs as, well as Langevin temperature coupling with a friction coefficient of 1 ps^−1^. Three independent runs of 120 ns were performed for each system, denoted as run1, run2, and run3. Snapshots from trajectories were recorded every 100 ps.

### Docking simulations

The binding sites and binding poses for dopamine, serotonin, and TBZ to the AlphaFold2 modeled VMAT2 conformer (AF-Q05940-F1-model_v4) and the present cryo-EM-resolved structure were assessed using AutoDock Vina^83^. Molecular structures of protonated dopamine and serotonin were adopted from the previous studies^84,85^. Docking simulations were carried out using a grid with dimensions set to 65 x 65 x 70 Å^3^ to encapsulate the entire structure of the transporter. The exhaustiveness of the simulation was set to 50, and the algorithm returned a maximum of 20 ligand binding poses.

### Computational data analysis

MD trajectory analysis was performed in VMD^86^. For each snapshot, the TBZ binding affinity was calculated using contact-based binding affinity predictor PRODIGY-LIG^87^. The binding pockets of VMAT2 were assessed using Essential Site Scanning Analysis (ESSA)^88^ and Fpocket^89^, implemented in ProDy 2.0^90^. Sequence conservation of VMAT2 was computed by the ConSurf web server using default parameters^91^.

## Supplementary Material

Supplement 1 **Extended Data Figure 1. Biochemical characterization, construct design, and sequence conservation of VMAT2. a,** SDS-PAGE gel showing purified VMAT2 chimera which migrates as a ~75 kDa species. b, Fluorescence-detection-size exclusion chromatography of purified VMAT2 chimera. The yellow trace is the fluorescence of mVenus and the black trace is of Trp. c, Time course accumulation of serotonin in vesicles using 1 µM ^3^H-serotonin for wild-type (black trace) and chimera (red trace). **d,** Sequence of VMAT2 colored by sequence variation across 150 VMAT2 sequences from different species, using Consurf server^91^. The position of mVenus and the GFP-Nb are indicated with arrows. Residues in EL1 that are not resolved in the cryo-EM map are also noted. **e,** VMAT2 model colored by sequence conservation.
**Extended Data Figure 2. Cryo-EM data processing of the VMAT2-tetrabenazine complex.** A representative micrograph (defocus −1.3 µm) is shown (scale bar equals 80 nm). The workflow depicts the data processing scheme used to reconstruct VMAT2. Two datasets were collected comprising 7,742 and 17,133 micrographs respectively. Movies were corrected for drift using patch motion correction and resultant micrographs were used to estimate defocus and pick particles. Blob picking followed by template picking was utilized to select approximately 5 million particles from each dataset. 2D classification was used to sort particles and the sorted particles were subjected to ab-initio reconstructions to obtain initial reference. Next, all of the particles picks from each dataset were subjected to multiple rounds of heterogeneous classification/refinement with the ab-initio VMAT2 map and two ‘decoy’ classes (yellow, a spherical blob and red, empty detergent micelle) starting with a box size of 128 pixels, followed by subsequent rounds of classification at box sizes of 256 and 384 pixels (full box size). This resulted in approximately 500k particles after combining both datasets. Particles underwent non-uniform refinement and further rounds of 2D classification and heterogeneous refinement to select particles with higher resolution features. Local refinements with a mask that excluded the detergent micelle further improved the resolution of the reconstruction. Non-uniform refinements, local refinement and CTF refinements with a mask focused on VMAT2 further improved the resolution. Bayesian polishing was utilized to correct local particle motion followed by further rounds of 2D classification, heterogeneous refinements, and CTF refinement. The final stack of 92k particles was then subjected to local refinement to produce the final unsharpened map. DeepEMhancer was used to locally sharpen the map for interpretation.
**Extended Data Figure 3. Cryo-EM maps and interpretation of VMAT2 reconstruction. a,** Cryo-EM density colored by local resolution estimation. **b,** FSC curves for cross-validation, the unmasked map (blue), loose mask (green), tight mask (red) and the reported corrected (purple) curves. The dotted line indicates an FSC value of 0.143. **c,** FSC curves for model versus half map 1 (working, red), half map 2 (free, blue) and model versus final map (black). **d,** Angular distribution of particles used in the final reconstruction. **e,** Cryo-EM density segments of TM1 to TM12.
**Extended Data Figure 4. Tetrabenazine docking and molecular dynamics simulations. a,** Time evolution of Cα root mean square deviations (RMSD) from the cryo-EM-resolved VMAT2 structure; and **b,** computed RMSD of TBZ, in three different runs. **c,** Histogram of TBZ binding affinities, summarized over all three runs. Binding affinities were calculated using PRODIGY-LIG applied to 800 evenly collected snapshots between 20 ns to 100 ns from each run. **d,** The TBZ binding poses and variations of W318 captured in the MD simulation runs 1–3, with a snapshot taken every 4 ns. The ligand conformations are shown in cyan sticks with blue stick illustrating cryo-EM resolved binding pose. The variations of W318 are displayed in purple sticks with dark purple showing the cryo-EM-resolved orientation. Docking simulations identified **e,** the most favorable (−9.7 kcal/mol) binding pose of TBZ, captured by run2. **f,** The TBZ pose (−9.3 kcal/mol) that closely resembles the cryo-EM-resolved structure (captured by runs 1,3). The RMSD from the resolved TBZ pose is 3.0 to 0.4 Å in **e** and **f**, respectively.
**Extended Data Figure 5. Point mutants in tetrabenazine binding site. a,** Binding site showing the positions of L37 and V232 which are a phenylalanine and a leucine in VMAT1 respectively. **b,** Plots of binding of 60 nM of [^3^H]dihydrotetrabenazine. The bars show the means and points show the value for each technical replicate. Error bars show the s.e.m. **c,** Sequence alignment of VMAT1, VMAT2, VAChT, and VPAT. The positions of mutated residues are shown boxed and in red. The positions of human variants are shown in blue boxes. d, Human variants of VMAT2, P316A, P237H and P387L localize to EL4 and the luminal ends of TM5 and TM9 respectively.
**Extended Data Figure 6. Comparison of the VMAT2-TBZ structure with the predicted Alphafold structure and with other MFS transporters. a,** Comparison of the cryo-EM structure (tan) vs. Alphafold (grey). The position of TMHs 1, 7, 8, 11 and EL4 in the cryo-EM structure show the most substantial differences and are shown for clarity. **b,** Comparison with the outward-open VGLUT2 structure (PDB code 8SBE) shown in blue. **c,** Comparison with the inward-open GLUT4 structure (7WSM) shown in magenta.
**Extended Data Figure 7. Docking-predicted binding poses of dopamine and serotonin. a**, Docking of dopamine and **b**, serotonin to the cryo-EM-resolved VMAT2. The most energetically favorable pose is shown; residues within 4 Å of the ligand are showed in sticks. Dopamine and serotonin are displayed in violet and cyan as van der waals (VDW) surfaces. The amine group of dopamine and serotonin is in close contact with E312 and their respective hydroxyl group(s) interact with R189.

Supplement 2 Extended Data Table 1. Cryo-EM data collection, refinement and validation statistics *Local resolution range at 0.5 FSC.
