# Supplementary material for: Structural mechanisms for VMAT2 inhibition by tetrabenazine
Source: bioRxiv. 2024 Feb 1:2023.09.05.556211. Originally published 2023 Sep 5. Preprint. [Version 2] doi: 10.1101/2023.09.05.556211 (PMC10508774; doi:10.1101/2023.09.05.556211)
Supplement: Supplement 1 — Figure Supplement 1. Biochemical characterization, construct design, and sequence conservation of VMAT2. a, The mVenus and GFP-Nb was fused the N- and C-terminus of VMAT2 and the length of the termini are varied to find constructs which can be studied by cryo-EM and retain functional activity. b, Sequence and secondary structure prediction. The position of the various transmembrane helices are shown and the position of mVenus and GFP-Nb. c, Screening of various constructs by FSEC. d, The SEC profile of the 17–481 chimera exhibits a single monodisperse peak. e, SDS-PAGE gel showing purified VMAT2 chimera which migrates as a ~75 kDa species. f, Time course accumulation of serotonin in vesicles using 1 μM 3H-serotonin for wild type (black trace) and chimera (red trace). g, VMAT2 colored by sequence variation from different species, using the Consurf server1. Figure Supplement 2. Cryo-EM data processing of the VMAT2-tetrabenazine complex. A representative micrograph (defocus −1.3 μm) is shown (scale bar equals 80 nm). The workflow depicts the data processing scheme used to reconstruct VMAT2. Two datasets were collected comprising 7,742 and 17,133 micrographs respectively. Movies were corrected for drift using patch motion correction in cryosparc64 and resultant micrographs were used to estimate defocus and pick particles. Blob picking followed by template picking was utilized to select approximately 5 million particles from each dataset. 2D classification was used to sort particles and the sorted particles were subjected to ab-initio reconstructions to obtain initial reference. Next, all of the particles picks from each dataset were subjected to multiple rounds of heterogeneous classification/refinement with the ab-initio VMAT2 map and two ‘decoy’ classes (yellow, a spherical blob and red, empty detergent micelle) starting with a box size of 128 pixels, followed by subsequent rounds of classification at box size of 256. This resulted in approximately 212k particles afte [file media-1.pdf]

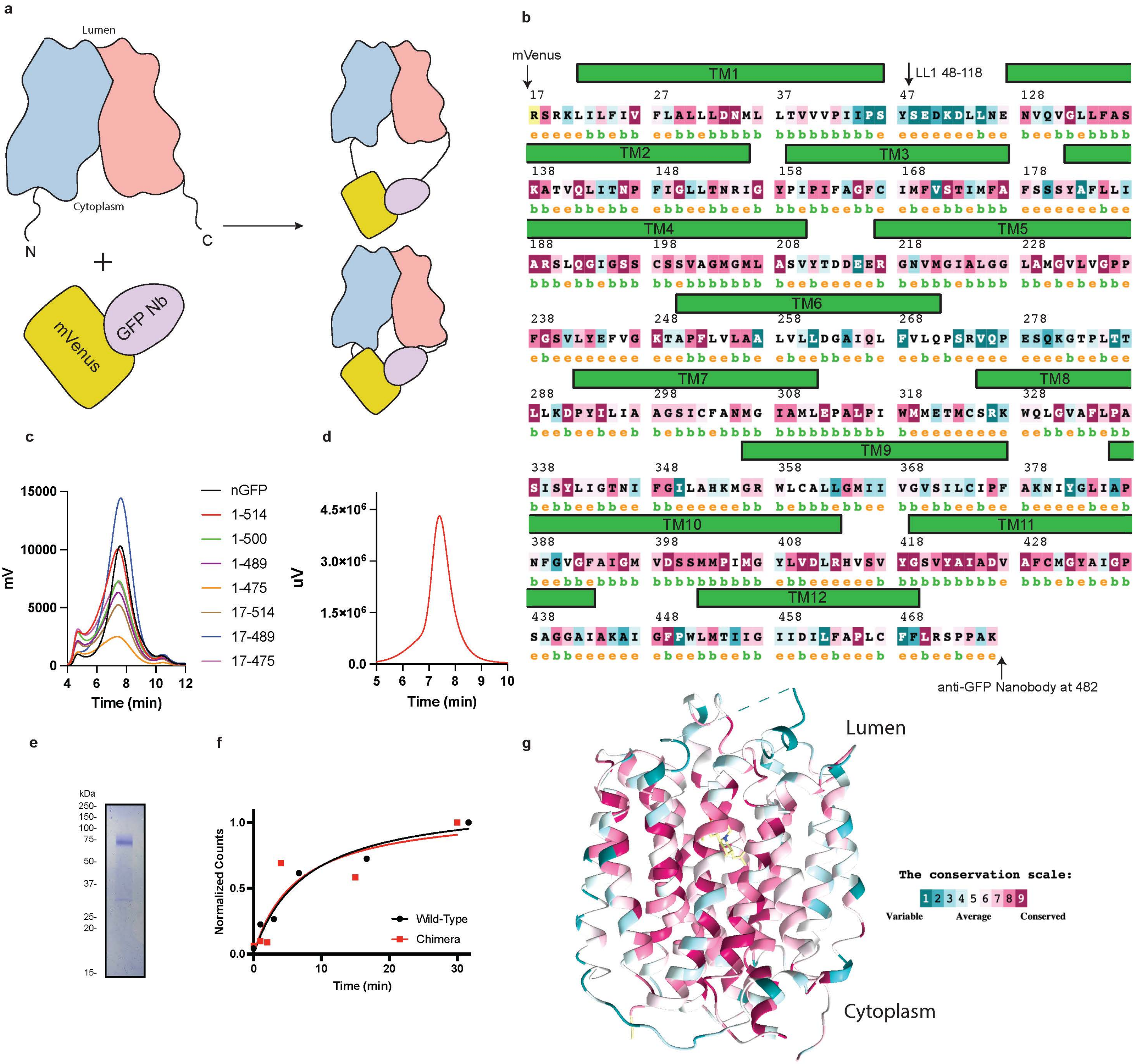

Supplementary Figure 1

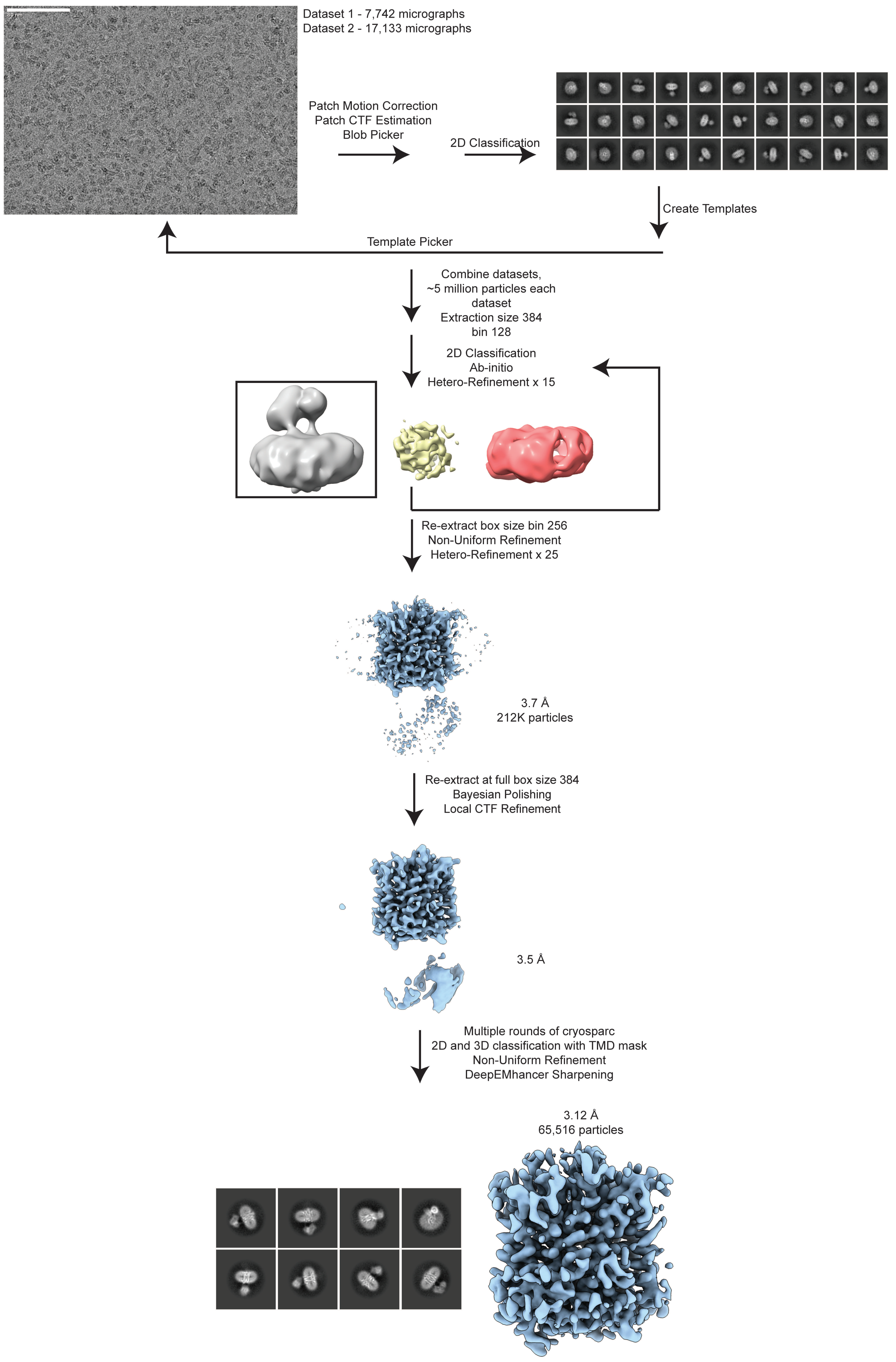

Supplementary Figure 2

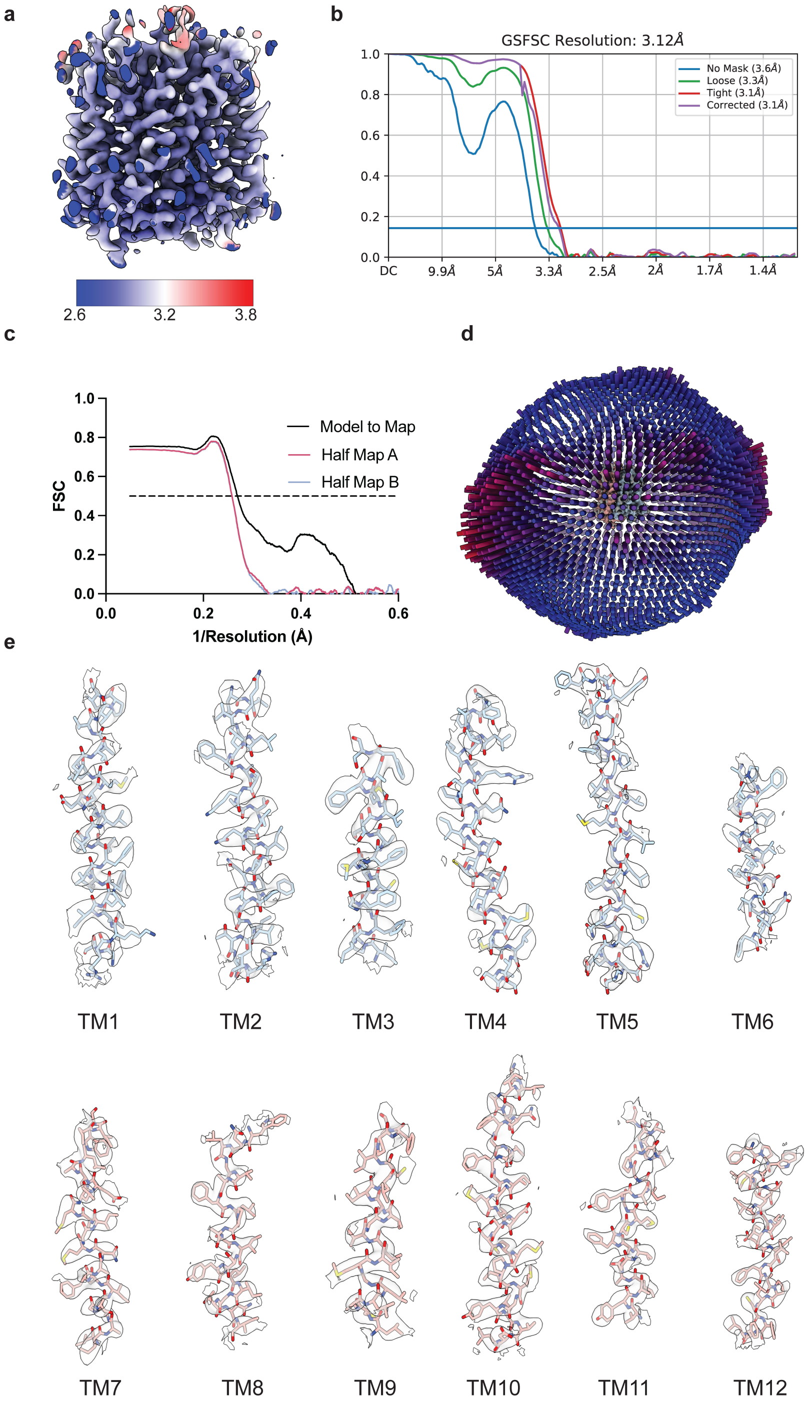

Supplementary Figure 3

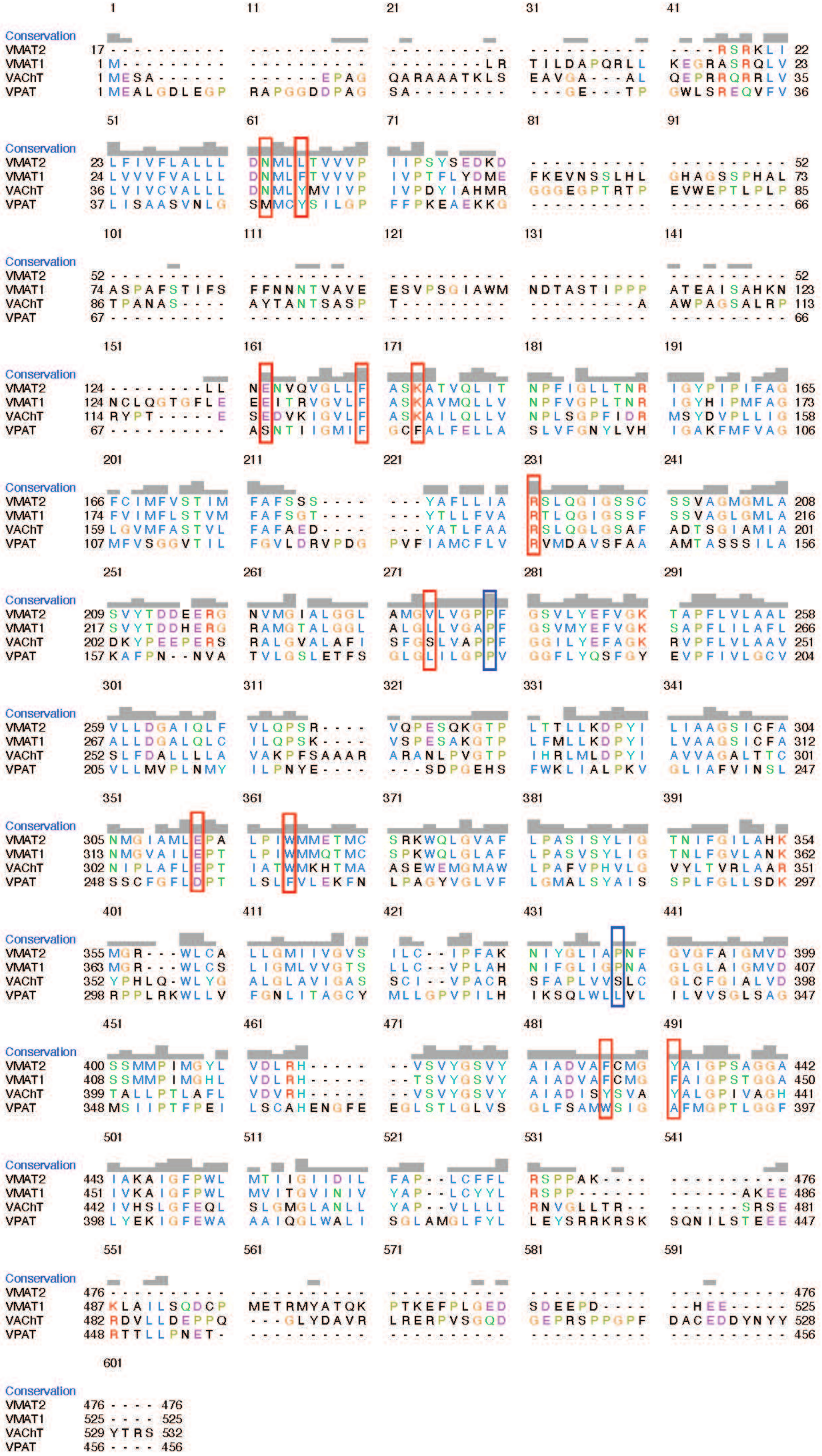

Supplementary Figure 4

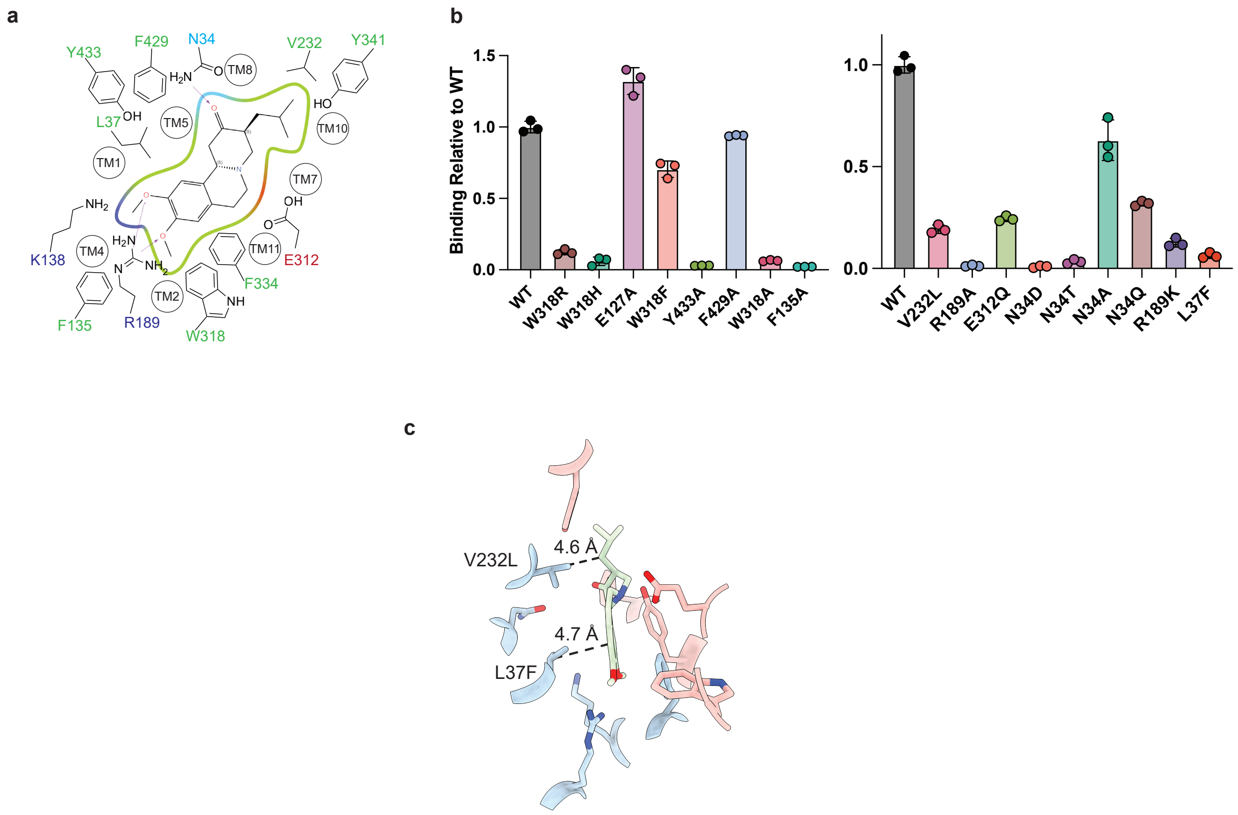

Supplementary Figure 5

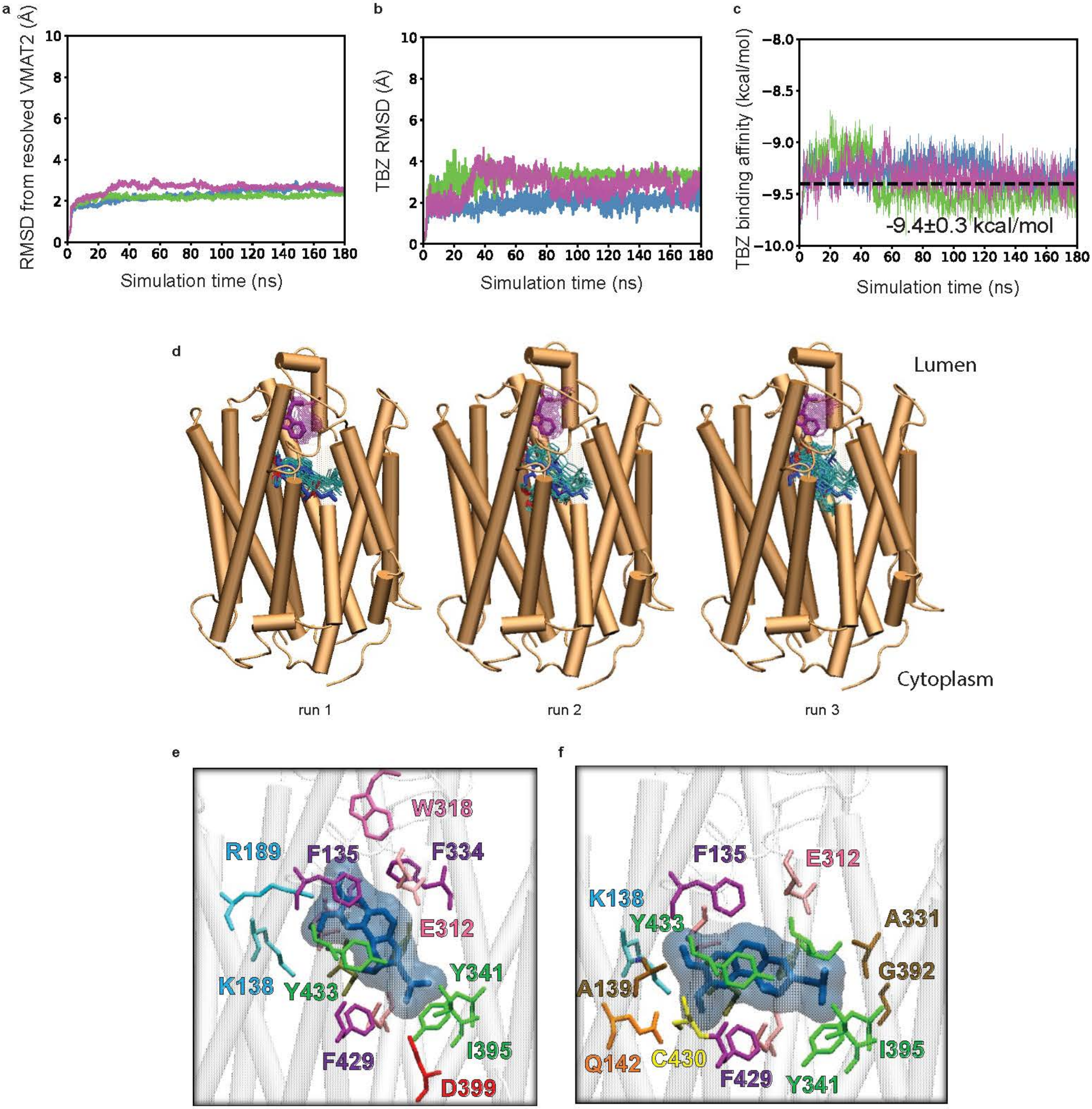

Supplementary Figure 6

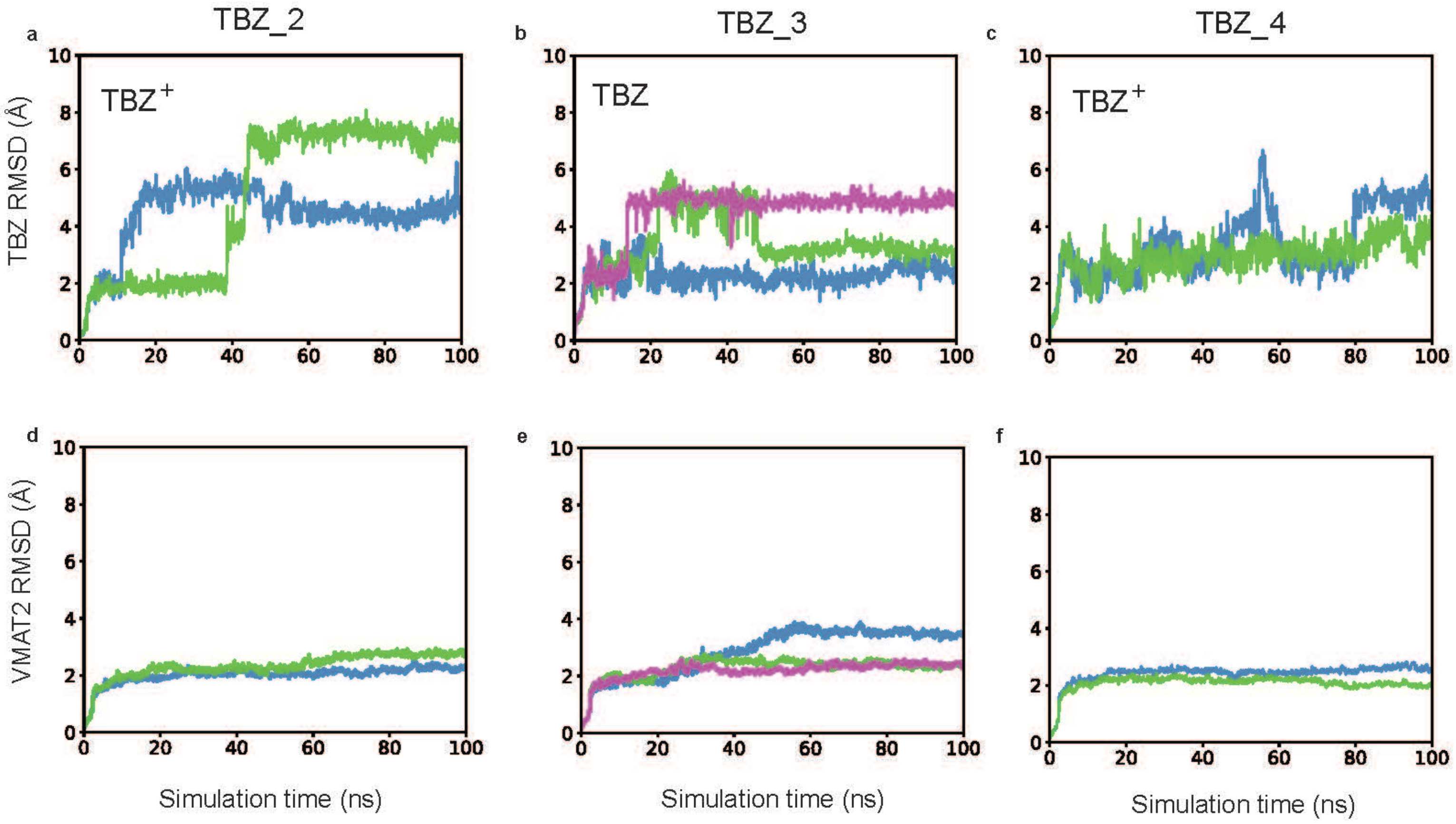

Supplementary Figure 7

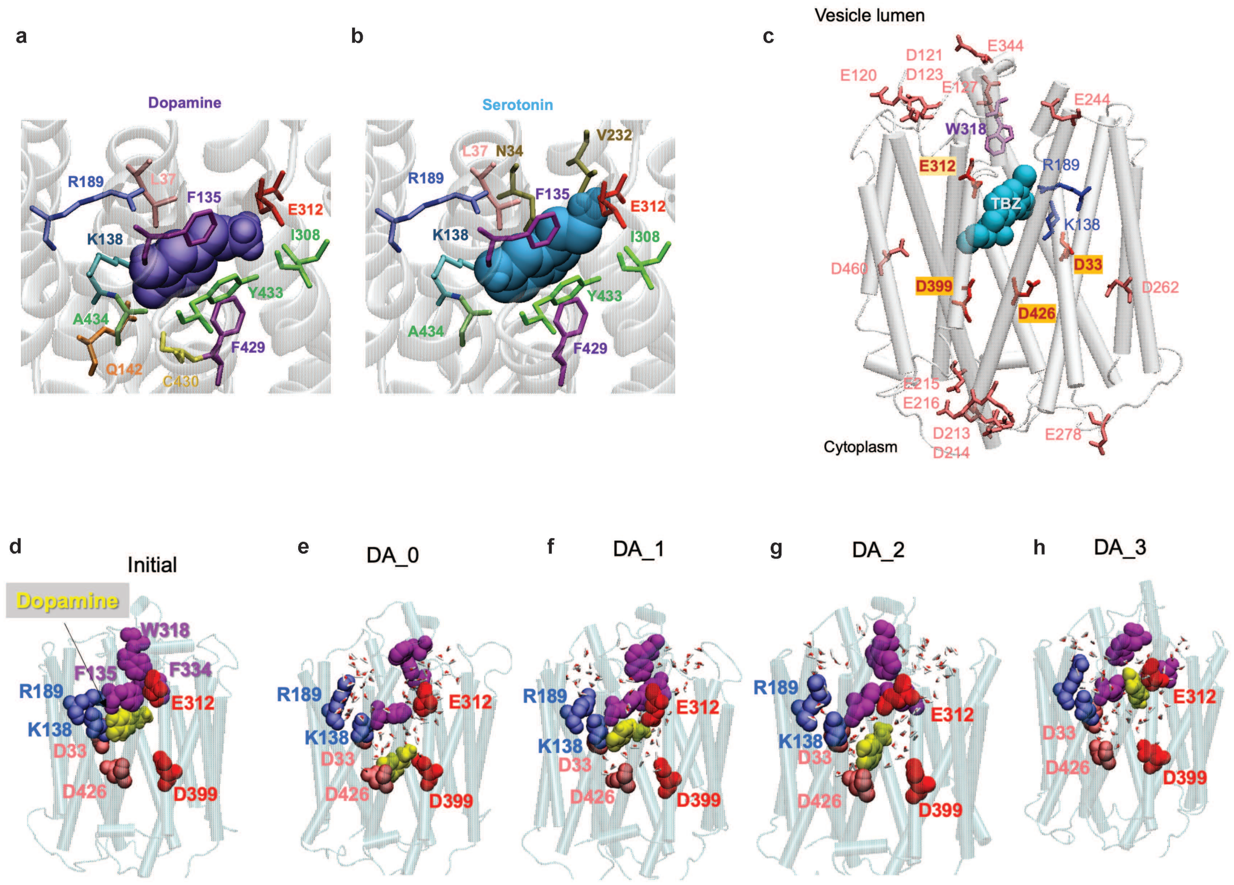

Supplementary Figure 8

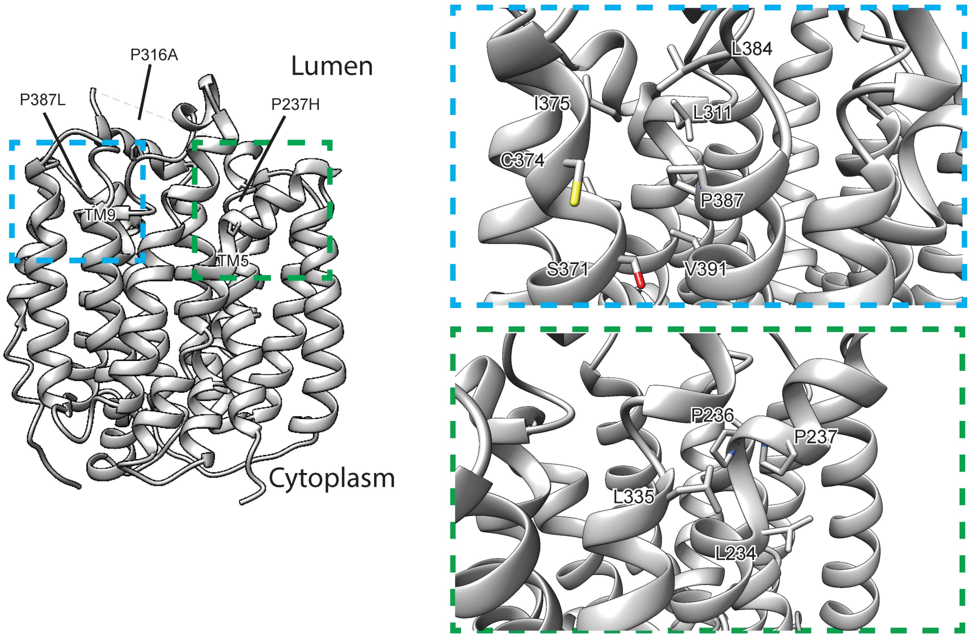

Supplementary Figure 9

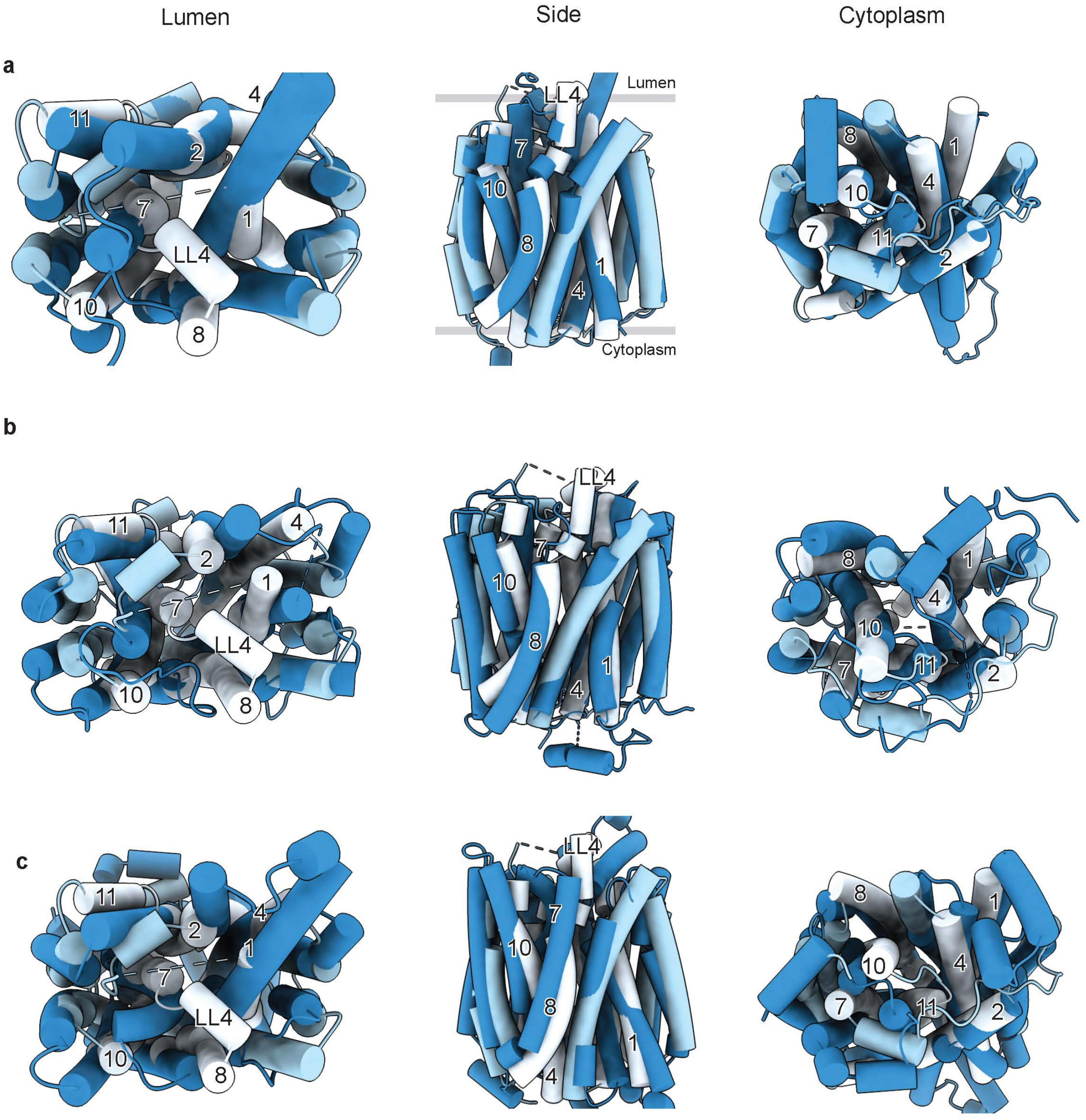

Supplementary Figure 10
