# Supplementary material for: Structural mechanisms for VMAT2 inhibition by tetrabenazine
Source: bioRxiv. 2024 Feb 1:2023.09.05.556211. Originally published 2023 Sep 5. Preprint. [Version 2] doi: 10.1101/2023.09.05.556211 (PMC10508774; doi:10.1101/2023.09.05.556211)
Supplement: Supplement 2 [file NIHPP2023.09.05.556211v2-supplement-2.pdf]

bioRxiv preprint doi: https://doi.org/10.1101/2023.09.05.556211; this version posted February 1, 2024. The copyright holder for this preprint (which was not certified by peer review) is the author/funder, who has granted bioRxiv a license to display the preprint in perpetuity. It is made available under aCC-BY 4.0 International license.



**Movie Supplement Legends**

**Movie Supplement 1** (related to Figure Supplement 6): **Movie_S1.mpg**. Binding and coordination of neutral TBZ (*cyan* van der Waals (vdW) representation observed in a 180 ns MD simulation (*run 1*). Three runs were performed for VMAT2 embedded in a lipid bilayer in the presence of TBZ, where E312 and D399 were protonated. We focus here on the time evolution of TBZ coordination, hence the naming of the runs as TBZ_1 run 1, TBZ_1 run 2, and TBZ_1 run 3. Movie S1 displays TBZ_1 run 1. For each snapshot, water molecules (in CPK format; ball and stick) within 4 Å of TBZ are shown; and acidic, basic, hydrophilic, and hydrophobic residues within 4 Å of TBZ are displayed in *red*, *blue, green* and *orange licorice* representations, respectively. The predominant pose adopted by TBZ is closely similar to that resolved in our cryo-EM structure, and illustrated in Figure Supplement 6 panel e. See Table 3 for the description of the runs.

**Movie Supplement 2** (related to Figure Supplement 6): **Movie_S2.mpg**. Binding and coordination of neutral TBZ (*cyan* vdW representation) observed in a 180 ns MD simulation (TBZ_1 run 2), in the same format as Movie Supplement 1. TBZ slightly altered its position within the same binding pocket, to stabilize the pose illustrated in Extended Data Figure 6 panel f.

**Movie Supplement 3** (related to Figure Supplement 6): **Movie_S3.mpg**. Binding and coordination of neutral TBZ (*cyan* van Der Waals (vdW) format) observed in a 180 ns MD simulation (TBZ_1 run 3), in the same format as Movie S1. TBZ adopts a pose similar to that resolved in the cryo-EM structure.

bioRxiv preprint doi: https://doi.org/10.1101/2023.09.05.556211; this version posted February 1, 2024. The copyright holder for this preprint (which was not certified by peer review) is the author/funder, who has granted bioRxiv a license to display the preprint in perpetuity. It is made available under aCC-BY 4.0 International license.



**Movie Supplement 4** (related to Table 3): **Movie_S4.mpg**. Same as Movie Supplement 1-3, with neutral TBZ, but with the additional protonation of D426 (TBZ_3 run1). TBZ adopted the predominant pose similar to that resolved in the cryo-EM structure.

**Movie Supplement 5** (related to Table 3): **Movie_S5.mpg**. MD simulation of the binding and coordination of protonated TBZ ($TBZ^+$; *pink* vdW format) to VMAT2 with protonated E312 and D399. This is a 100 ns run, termed TBZ_2 run 1. The same format as Movie Supplement 1-3 is adopted for the coordinating residues. TBZ tends to alter its binding pose to approximate the one observed in Movie Supplement 2.

**Movie Supplement 6** (related to Table 3): **Movie_S6.mpg**. Same as Movie Supplement 4, except for the protonation of TBZ ($TBZ^+$ shown in pink vdW representation). This is a 100 ns run, termed TBZ_4 run 1. In the presence of protonation, TBZ preferentially samples the pose observed in Movie Supplement 2 and 5.

**Movie Supplement 7** (related to Table 5): **Movie_S7.mpg**. Fluctuations in dopamine binding pose (DA; *yellow* vdW spheres) within its binding pocket, observed in a 100 ns MD simulation of VMAT2 in the presence of DA (system DA_2 in Table 5). VMAT2 E312 and D399 are protonated; and D33 and D426 are deprotonated. Hydrophobic gate residues F135, W318 and F334 are displayed in *purple* vdW spheres, and acidic and basic residues K138 and R189 are shown in *red* and *blue* vdW representation.

**Movie Supplement 8** (related to Table 5: **Movie_S8.mpg**. Release of dopamine (DA; *yellow* vdW spheres) from the binding pocket, observed in a 100 ns MD simulation (system DA_3 in Table 4). E312, D399 and D426 were protonated and D33 was deprotonated.

**Movie Supplement 9** (related to Figure 5): **Movie_S9.mpg**. Release of DA to the vesicular lumen (*yellow* VDW spheres), observed after protonating D33, E312, D399 and D426 (*red* vdW spheres). The movie represents a 200 ns MD simulation of system DA_4 in Table 5.
