# Supplementary material for: Structural Mechanisms for VMAT2 inhibition by tetrabenazine
Source: bioRxiv. 2023 Sep 5:2023.09.05.556211. Preprint. [Version 1] doi: 10.1101/2023.09.05.556211 (PMC10508774; doi:10.1101/2023.09.05.556211)
Supplement: Supplementary file 1 [file media-1.pdf]

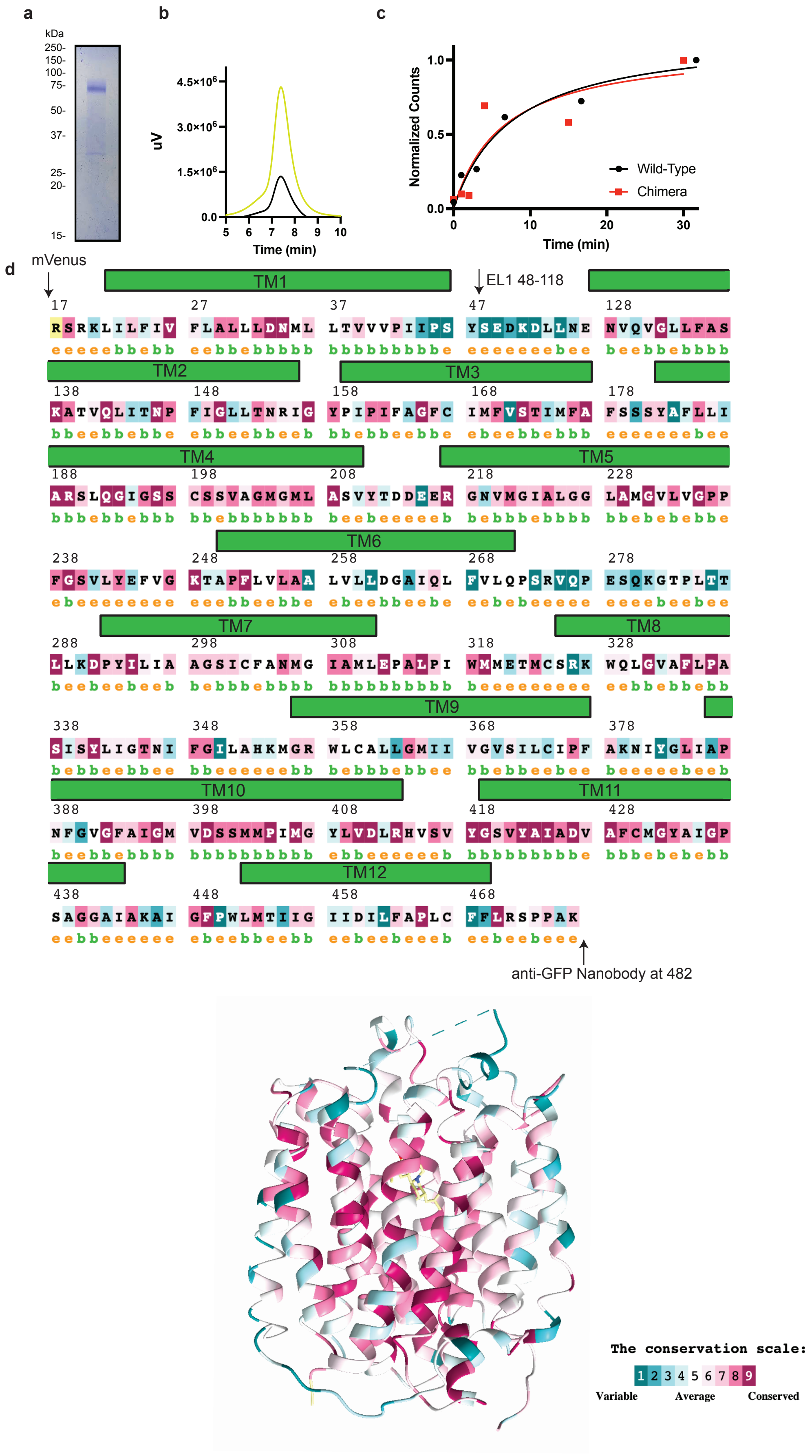

Extended Data Figure 1

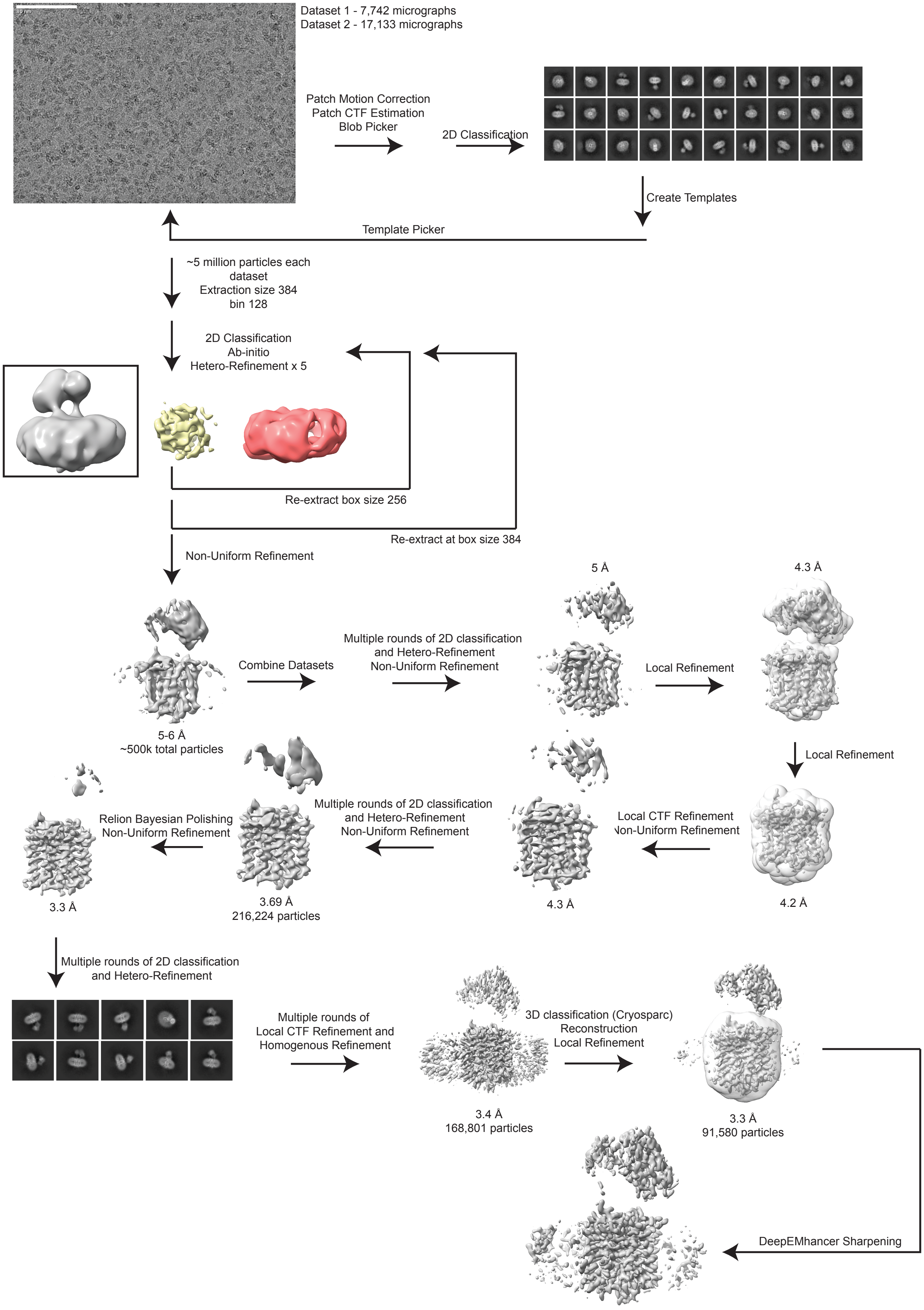

Extended Data Figure 2

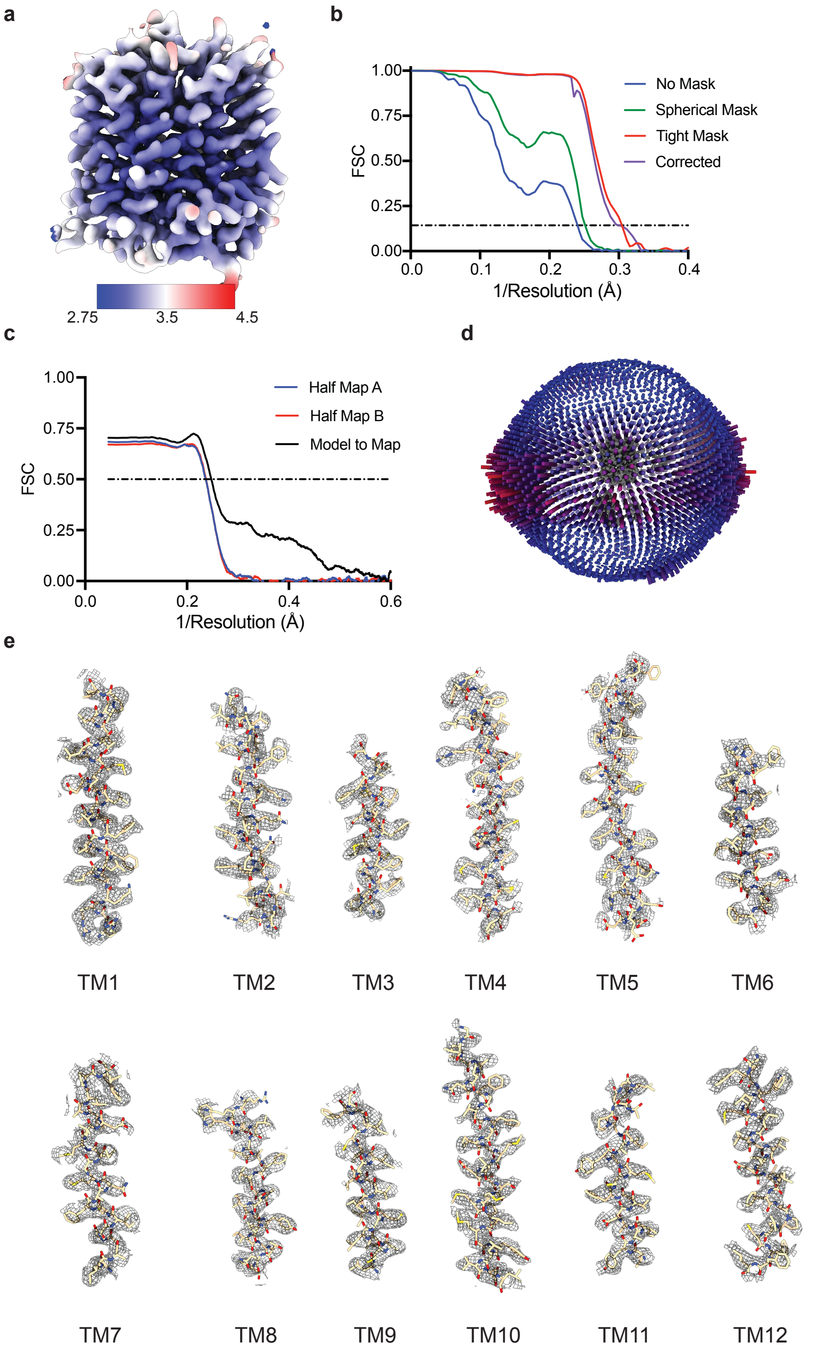

Extended Data Figure 3

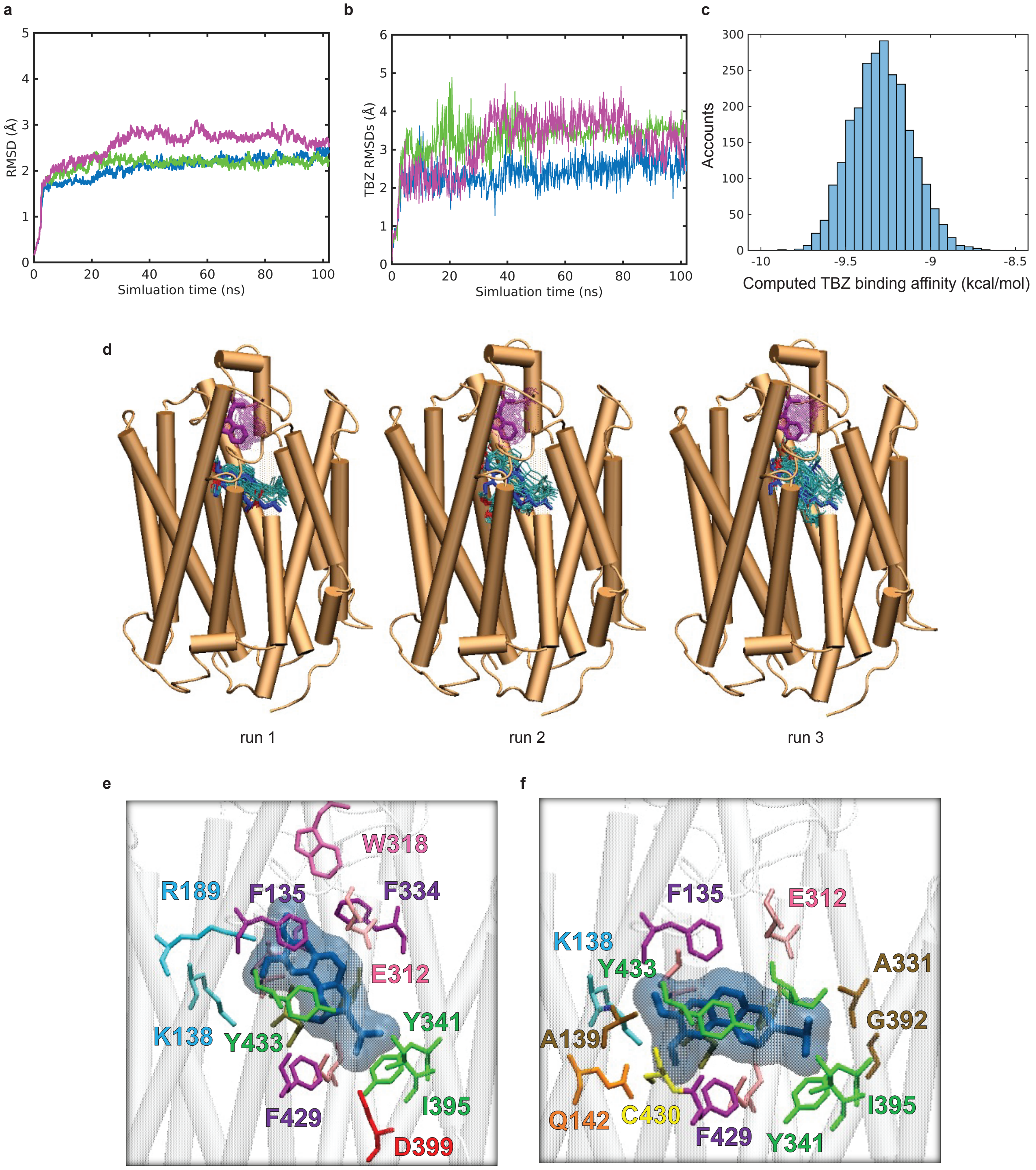

Extended Data Figure 4

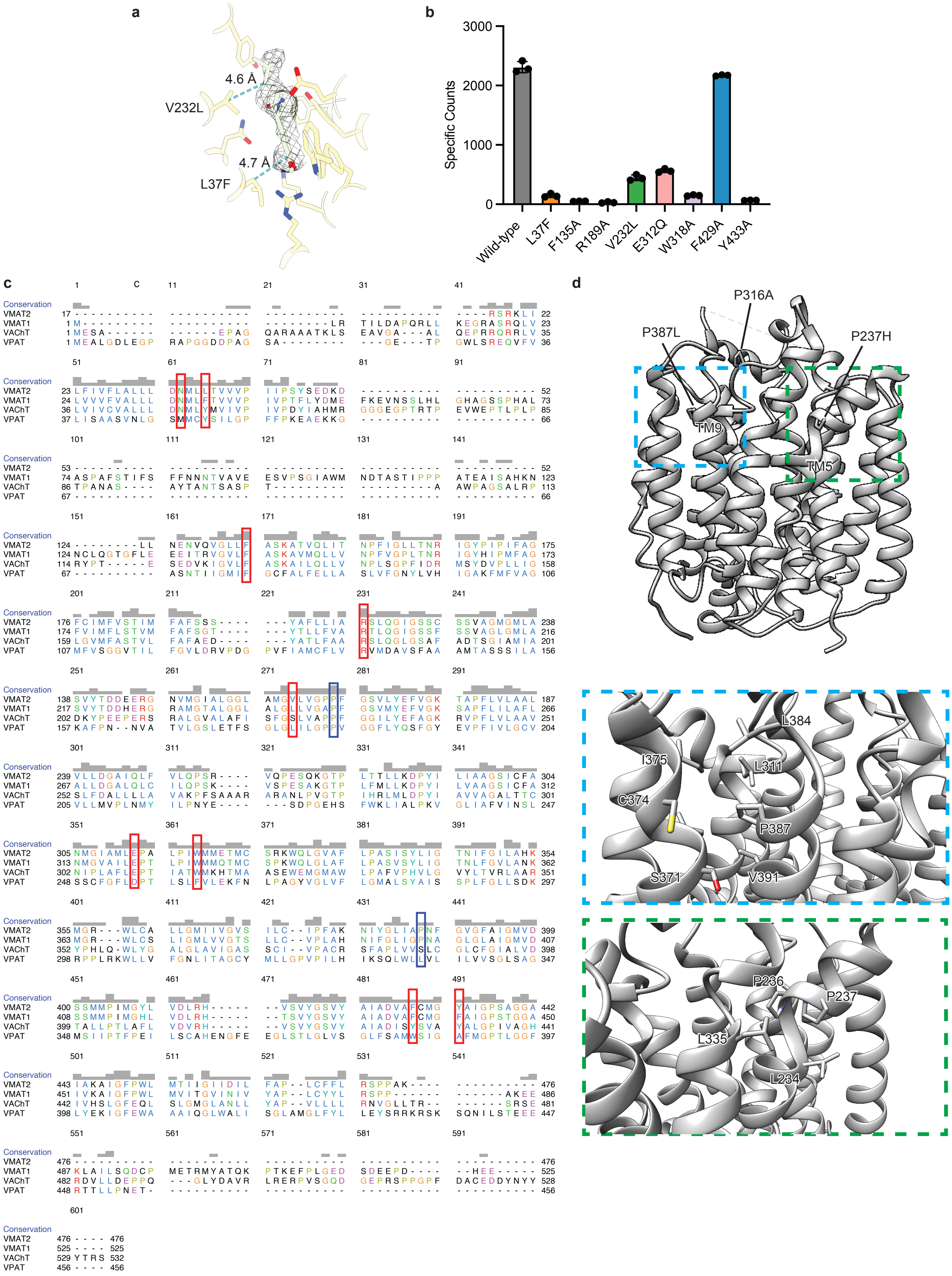

Extended Data Figure 5

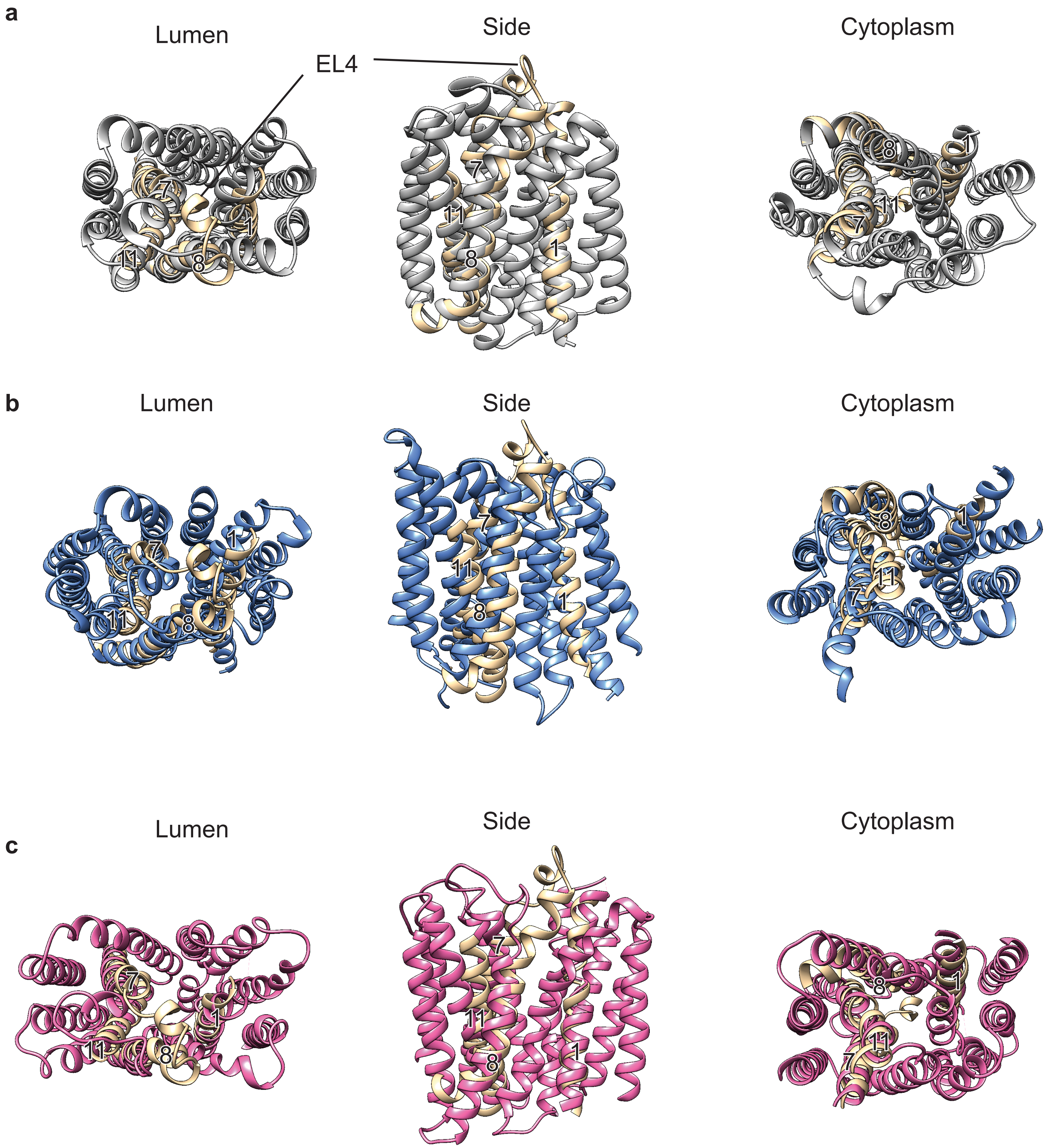

Extended Data Figure 6

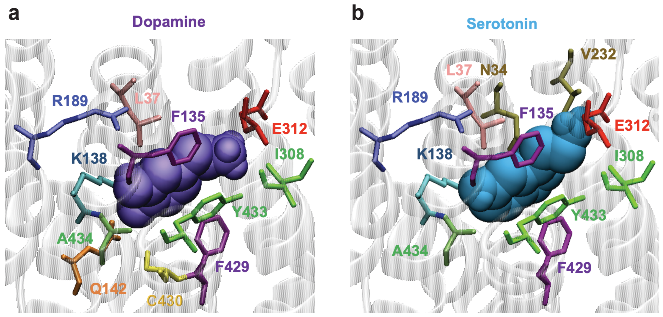

Extended Data Figure 7
