# Supplementary material for: Structural Mechanisms for VMAT2 inhibition by tetrabenazine
Source: bioRxiv. 2023 Sep 5:2023.09.05.556211. Preprint. [Version 1] doi: 10.1101/2023.09.05.556211 (PMC10508774; doi:10.1101/2023.09.05.556211)
Supplement: Supplementary file 2 [file media-2.pdf]

	VMAT2-TBZ (EMDB-41269) (PDB 8THR)
**Data collection and processing**	
Magnification	77,279
Voltage (kV)	300
Electron exposure (e–/Å^2)	60
Defocus range (μm)	-1.0 – -2.5
Pixel size (Å)	0.647
Symmetry imposed	C1
Initial particle images (no.)	~10,000,000
Final particle images (no.)	91,580
Map resolution (Å)	3.3
FSC threshold	0.143
Map resolution range (Å)*	34.2 - 3.0
**Refinement**	
Initial model used (PDB code)	
Model resolution (Å)	4.0
FSC threshold	0.5
Model resolution range (Å)	17.9 - 4.0
Map sharpening *B* factor (Å^2)	
Model composition	
Non-hydrogen atoms	2937
Protein residues	389
Ligands	1
B factors (Å^2)	
Protein	57.8
Ligand	57.5
R.m.s. deviations	
Bond lengths (Å)	.0004 (0)
Bond angles (°)	.739 (1)
Validation	
MolProbity score	1.9
Clashscore	9.02
Poor rotamers (%)	0.64
Ramachandran plot	
Favored (%)	93.77
Allowed (%)	6.23
Disallowed (%)	0.00
